# METTL family proteins: functional heterogeneity, tumor regulatory networks, immune microenvironment remodeling and therapeutic potential

**DOI:** 10.3389/fimmu.2026.1952952

**Published:** 2026-10-14

**Authors:** Qiujie Ding, Peng Wang, Hailong Yu, Zixue Xuan, Chao Yang, Kai Wang

**Affiliations:** 1 Key Laboratory of Epigenetics and Oncology, Research Center for Preclinical Medicine, Southwest Medical University, Luzhou, China; 2 School of Basic Medical Sciences, Southwest Medical University, Luzhou, China; 3 The Affiliated Traditional Chinese Medicine Hospital, Southwest Medical University, Luzhou, China; 4 Center for Clinical Pharmacy, Cancer Center, Department of Pharmacy, Zhejiang Provincial People’s Hospital (Affiliated People’s Hospital), Hangzhou, China; 5 National Engineering Research Center for Marine Aquaculture, Institute of Innovation & Application, Zhejiang Ocean University, Zhoushan, China

**Keywords:** antitumor, immune response, m6A modification, methyltransferase, METTL family, METTL inhibitor

## Abstract

RNA methylation serves as an essential post-transcriptional epigenetic modification for gene expression regulation. The methyltransferase-like (METTL) family is characterized by conserved methyltransferase domains. These members can recognize multiple RNA substrates including messenger RNA (mRNA), ribosomal RNA (rRNA), and mitochondrial RNA (mtRNA), and engage in a variety of RNA methylation processes. They exhibit markedly heterogeneous tumor-regulatory functions. The METTL3/14 complex regulates tumor proliferation, metastasis, metabolic reprogramming and immunotherapy resistance by catalyzing mRNA N6-methyladenosine (m6A) modification. METTL5 targets rRNA and drives translational reprogramming to participate in tumor progression and T cell immune resistance. METTL17 mediates multiple methylations of mtRNA to deregulate mitochondrial metabolism and drive malignant tumor phenotypes. METTL16 possesses both mRNA m6A catalytic and small nuclear RNA non-catalytic functions, and participates in tumor progression and immune escape. METTL4 catalyzes N6, 2’-O-dimethyladenosine methylation to modulate target gene translation and facilitate tumorigenesis. Beyond RNA methylation, METTL7A/B promotes resistance to tumor-targeted drugs via m6A-independent mechanisms of DNA binding and protein interactions. Notably, the same METTL protein yields distinct immunoregulatory effects in tumor cells, T cells, macrophages and natural killer cells, with obvious context-dependence. In this paper, we systematically summarize structural features and functions of METTL family proteins, together with mechanisms underlying their regulation of the tumor immune microenvironment. We also consolidate the research progress and transformation bottlenecks of multiple targeted candidate molecules. It provides a theoretical basis and new ideas for further investigations of METTL-based tumor immunotargeted therapy.

## Introduction

Methyltransferases are pivotal enzymes that catalyze methylation modifications of various substrates, including RNA, DNA, and proteins (1–5), significantly impacting tumorigenesis, survival, metastasis, and immune evasion (6–12). Based on substrate specificity, methyltransferases are categorized into RNA methyltransferases, DNA methyltransferases, and histone methyltransferases, with RNA methyltransferases serving as primary enzymes that mediate epigenetic modifications of the transcriptome and regulate RNA metabolism in tumors (13, 14).

The methyltransferase-like (METTL) family represents a highly functionally diverse superfamily within RNA methyltransferase-related proteins (15). Members possess methyltransferase domains (MTDs) capable of recognizing various RNA substrates and participating in the modification processes of messenger RNA (mRNA), transfer RNA (tRNA), ribosomal RNA (rRNA), and other RNAs (15, 16). However, heterogeneity among METTL family members in terms of RNA substrate preference, modification types, catalytic activity, auxiliary functions, subcellular localization, and overall cellular roles complicates the classification of all METTL proteins as m6A methyltransferases. This review intentionally narrows its focus to those METTL members extensively studied in relation to tumor regulation and the immune microenvironment, previously categorized as m6A-related METTLs, including METTL3, METTL14, METTL4, METTL5, METTL7A/B, METTL16, and METTL17 (15).

These proteins do not form a functionally homogeneous subgroup of m6A writers. The METTL3/14 complex is recognized as a classic heterodimer responsible for m6A modifications of mRNAs and non-coding RNAs (ncRNAs) (17–22). METTL3 provides the core catalytic activity, while METTL14 serves mainly in structural scaffolding and substrate recognition, lacking independent m6A catalytic activity (23, 24). METTL16 is acknowledged as an m6A writer that recognizes specific RNA substrates, catalyzing m6A modification and displaying biological functions independent of its methyltransferase activity (25–29). METTL5 primarily facilitates rRNA methylation to enhance ribosome translation efficiency (30). METTL17 is primarily involved in mitochondrial RNA (mtRNA) modification and mitochondrial metabolism, although the exact modification types and catalytic mechanisms remain to be fully defined (31). While METTL4 and METTL7A/B target specific RNA substrates, their catalytic activity, substrate range, and dependency on m6A are context-dependent, with some functions potentially occurring through m6A-independent pathways (32–36). For comparative analysis, **Table 1** systematically summarizes the primary RNA substrates, catalytic modification categories, catalytic/accessory functions, known cofactors, subcellular localization, functions relevant to tumors and immunity, strength of evidence, and source of evidence (tumor cells, immune cells, or both).

**Table 1:** Summary of substrate preference, modification types, cellular functions and tumor immune roles of a subset of METTL family members.

METTL member	Principal RNA substrates	Catalytic modification categories	Catalytic/accessory functions	Known cofactors	Experimentally validated subcellular localization	Tumor-relevant functions	Immune-relevant functions	Strength of evidence^a^	Source of evidence (tumor cells, immune cells, or both)
METTL3	- experimentally validated substrates: mRNA (43) - reported substrates: mRNA (16), tRNA (16), rRNA (16), lncRNA (16) - indirect associations: circRNA (44), lncRNA (45, 46), pri-miRNA (47)	m6A (16, 43–46, 48)	catalytic (16, 43–46, 48)	METTL14 (49), WTAP (49), VIRMA (50), RBM15/15B (50, 51), ZC3H13 (50, 51), S-adenosylmethionine (52)	nucleus (40, 53, 54)	tumor promotion (44–46, 55–57), tumor inhibition (58)	inhibit CD8+ T cell function (59–62), maintain the immunosuppressive function of Tregs (63), participate in the differentiation and functional regulation of Th17 cells (64), upregulate PD-L1 expression (65–67), downregulate PD-L1 expression (60, 68), promote macrophage M2 polarization (38, 69–71), enhance the tumor-killing capacity of NK cells (72)	★★★	both
METTL4	- experimentally validated substrates: snRNA (73, 74) - reported substrates: mRNA (16), snRNA (16), miRNA (16) - indirect associations: mRNA (75), miRNA (34)	m6A (34, 73, 75), m6Am (36)	catalytic (34, 36, 73, 75)	S-adenosylmethionine (76)	nucleus (74, 77), mitochondria (78)	tumor promotion (36, 75)	unknown	★★	tumor cells
METTL5	- experimentally validated substrates (79, 80): - reported substrates: rRNA (16) - indirect associations: mRNA (81), rRNA (79, 82)	m6A (79–83)	catalytic (79–83)	TRMT112 (84), S-adenosylmethionine (84)	cytoplasm (30), nucleus (30)	tumor promotion (81, 85)	inhibit CD8+ T cell function (11, 86), upregulate PD-L1 expression (87)	★★	both
METTL7A/B	- experimentally validated substrates: not yet reported - reported substrates: lncRNA (42), mRNA (42) - indirect associations: mRNA (35, 88, 89)	m6A (15, 35, 88)	catalytic (15, 35, 88)	unknown	endoplasmic reticulum (90, 91), lipid droplet (91)	tumor promotion (92)	attenuate PD-L1-driven T cell immunosuppression (93), inhibit macrophage M2 polarization (94)	★★	both
METTL14	- experimentally validated substrates: mRNA (95, 96) - reported substrates: mRNA (16, 97) , tRNA (16), rRNA (16), lncRNA (16) - indirect associations: mRNA (98)	m6A (16, 95–98)	accessory (23, 24)	METTL3 (99), WTAP (100, 101)	nucleus (53, 54)	tumor inhibition (102), tumor inhibition (96, 97)	inhibit CD8+ T cell function (103–105), induce Treg differentiation and maintain their immunosuppressive function (106, 107), upregulate PD-L1 expression (108, 109), downregulate PD-L1 expression (110), weaken the tumor-clearing ability of macrophages (111), enhance the tumor-killing capacity of NK cells (72)	★★★	both
METTL16	- experimentally validated substrates: snRNA (27) - reported substrates: mRNA (16), rRNA (16), lncRNA (16), mtRNA (16), snRNA (16) - indirect associations: mRNA (112), lncRNA (112)	m6A (27, 112, 113)	catalytic (27, 112, 113)	S-adenosylmethionine (114)	nucleus (nucleolus) (115, 116), cytoplasm (116)	tumor promotion (29, 112, 117, 118), tumor inhibition (42, 119, 120)	downregulate PD-L1 expression (121), promote macrophage M2 polarization (38, 71)	★★	both
METTL17	- experimentally validated substrates: mt-rRNA (122) - reported substrates: mt-rRNA (42) - indirect associations: mtRNA (31)	m6A (31), m3C (31), m4C (31, 122), m5C (31, 122), m7G (31)	catalytic (31, 122)	Fe-S cluster (123), METTL15 (124)	mitochondria (31)	tumor promotion (31, 122)	unknown	★★	tumor cells

^a^Strength of evidence, ★★★, *In vitro* and *in vivo* experiments; ★★, *In vitro* experiments; ★, Correlation and bioinformatic prediction only.

Numerous studies indicate that the aforementioned METTL family members are involved in pathophysiological processes such as tumor progression, metastasis, chemoresistance, and immune evasion, primarily through the regulation of RNA methylation modifications or non-catalytic functions (16, 37–40). This area has emerged as a significant focus in cancer epigenetics (41, 42). For this review, literature was predominantly gathered from the PubMed, Web of Science, and Google Scholar databases. Key search terms included METTL family, m6A, RNA methylation, tumor, immune response, and METTL inhibitor, with a restriction to articles published within the past five years. The inclusion criteria for screened literature were: 1) original research papers and high-quality reviews; 2) studies addressing the structure, RNA modification, tumor biology, tumor immunology, and targeted drug development related to the specified METTL members. The final literature search was conducted on August 10, 2026. This review begins with a systematic overview of the structures and functions of METTL family proteins, followed by an examination of their multifaceted regulatory roles in tumors. Additionally, it summarizes their regulatory mechanisms pertaining to immune cells within the tumor microenvironment (TME). Emphasis is placed on their potential utility as tumor biomarkers and therapeutic targets, while also identifying current challenges and proposed future development directions. The primary objective of this review is to establish a theoretical framework for the development of novel tumor treatment strategies aimed at overcoming clinical acquired resistance.

In comparison to previous reviews on the METTL family and m6A, this review presents several distinctive features. First, it systematically differentiates between classical m6A writers, non-catalytic scaffold components, and other RNA methyltransferases, emphasizing the divergence between m6A-dependent and m6A-independent biological functions of METTL members. Second, it highlights METTL-mediated bidirectional immune regulation in tumor and immune cells, considering subcellular compartment specificity and analyzing context-dependent factors that may produce conflicting experimental outcomes. Third, it objectively evaluates the translational challenges faced by candidate METTL inhibitors, including selectivity, hematopoietic immunotoxicity, therapeutic window, drug resistance, and long-term safety. Finally, it highlights TME spatial heterogeneity and discusses the prospects for utilizing single-cell and spatial transcriptomics to elucidate METTL regulatory networks.

## Structure and functions of METTL family proteins

Human METTL proteins predominantly feature a conserved methyltransferase domain (MTD), with certain proteins also displaying unique N-terminal and C-terminal sequence structures (**Figure 1**).

**Figure 1:**
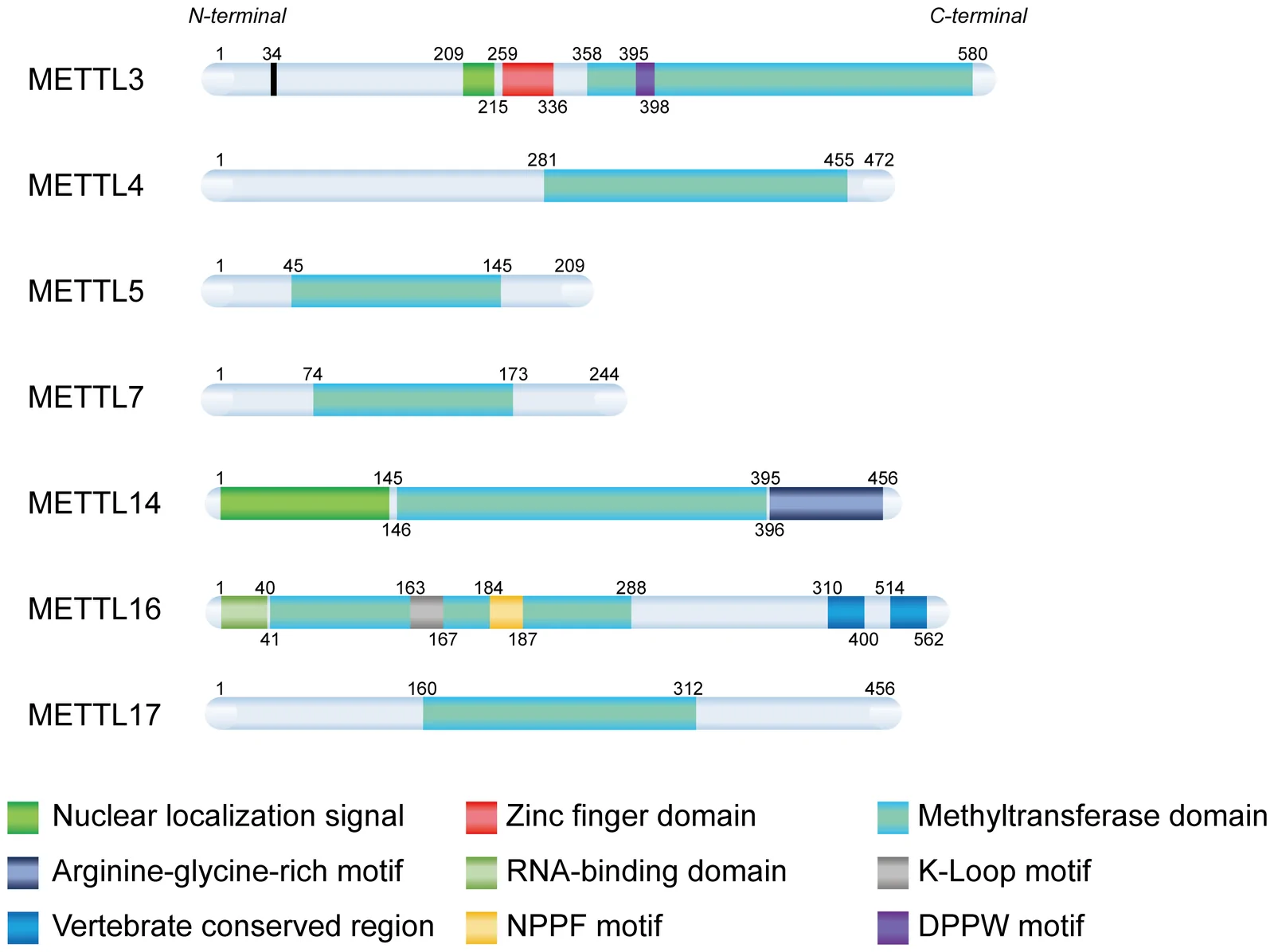
Structures of a subset of human METTL family proteins.

The MTD serves as the core catalytic domain for most METTL family proteins (15, 25, 27, 125–127). This domain typically adopts a conserved parallel seven-β-strand (7βS) structure, a classic conformation critical for mediating methylation functions. Functional elements within the MTD, including the NPPF motif, the DPPW motif, active site loop, K-loop, and substrate-binding loop (SBL), play critical roles in catalytic activity and substrate RNA binding. However, these elements are not universally conserved across the METTL family, and some members are predicted only at the sequence level, lacking experimental verification in structural biology. For instance, in the MTD of METTL3, a conserved DPPW motif (residues 395-398) exists, with Asp395 being essential for S-adenosylmethionine (SAM) binding and catalytic function, and Trp398 interacting with the methylated adenine through π-π stacking (125). In contrast, METTL14’s originally conserved DPPW motif has mutated to EPPL, resulting in the loss of critical amino acids necessary for SAM binding and leading to a complete loss of catalytic activity (125). The MTD of METTL5 contains three conserved regions vital for methyltransferase activity: the active site loop, SBL, and the catalytic loop with the NPPF motif (128). The integrity of the NPPF motif is essential for enzyme activity; experimental mutations indicate that substituting the terminal residues (Asn126-Phe129) with alanine directly diminishes METTL5’s catalytic capability (128). Its SBL, characterized by hydrophobic and positively charged basic residues (e.g., Tyr190-KFHKK-Lys196), stabilizes the substrate-binding pocket, while the positively charged side chains interact electrostatically with the phosphate backbone of rRNA, facilitating substrate recognition (128). METTL7A/B display a typical SAM-dependent methyltransferase conformation (90, 129, 130). Mutation analyses confirm that replacing the conserved aspartic acid in METTL7B with alanine entirely disrupts its methyltransferase activity (130). However, no high-resolution 3D structures of METTL7A/B have been experimentally resolved, leaving their substrate-recognition patterns and complete catalytic mechanisms yet to be elucidated. The NPPF motif (residues 184-187) and K-loop (residues 163-167) within the METTL16 MTD mediate its catalytic function, with residues Pro186 and Phe187 anchoring the target adenine base and enhancing substrate base conformational stability (114). The K-loop, positioned between the β3 and β4 strands, serves as a unique self-regulatory element. In the absence of RNA substrate binding, the K-loop adopts an α-helical conformation with the internal Lys163 occupying the SAM binding pocket, thus exerting an inhibitory effect. Upon RNA binding, the K-loop transitions to a loop structure, freeing the SAM binding site and activating METTL16’s catalytic activity (131). METTL17 features a canonical MTD structure (132) that regulates mitochondrial ribosome function in a SAM-dependent manner (133). Further validation through high-resolution structural analyses and *in vitro* enzymatic experiments is necessary to determine whether its diverse RNA-modifying effects arise from indirect regulation or intrinsic direct catalysis. Although METTL4 is predicted to harbor the MTD domain, supporting structural biological evidence remains absent.

The N-terminal sequences of METTL3/14 and METTL16 have been experimentally confirmed to play roles in RNA-substrate binding and determining protein subcellular localization (53, 127, 134, 135). The C-terminal region of METTL14 acts as the primary RNA-binding area that specifically recognizes and binds RNA G-quadruplex (rG4) structures, subsequently recruiting the METTL3/14 complex to the DRACH methylation sites adjacent to rG4 (136), thereby preserving the m6A methylation functionality of the complex (137). The vertebrate-conserved region (VCR), a METTL16-specific C-terminal sequence, facilitates its interaction with small nuclear RNA (snRNA) (27). Nonetheless, the precise function of this domain remains to be elucidated.

## Regulatory roles of METTL family proteins in tumors

Increasing evidence reveals that the aforementioned METTL family proteins regulate tumor cell growth, progression, and drug resistance through various mechanisms, including post-transcriptional regulation (RNA stability, processing, and translation), chromatin remodeling, DNA damage response, and post-translational modification (**Figure 2**).

**Figure 2:**
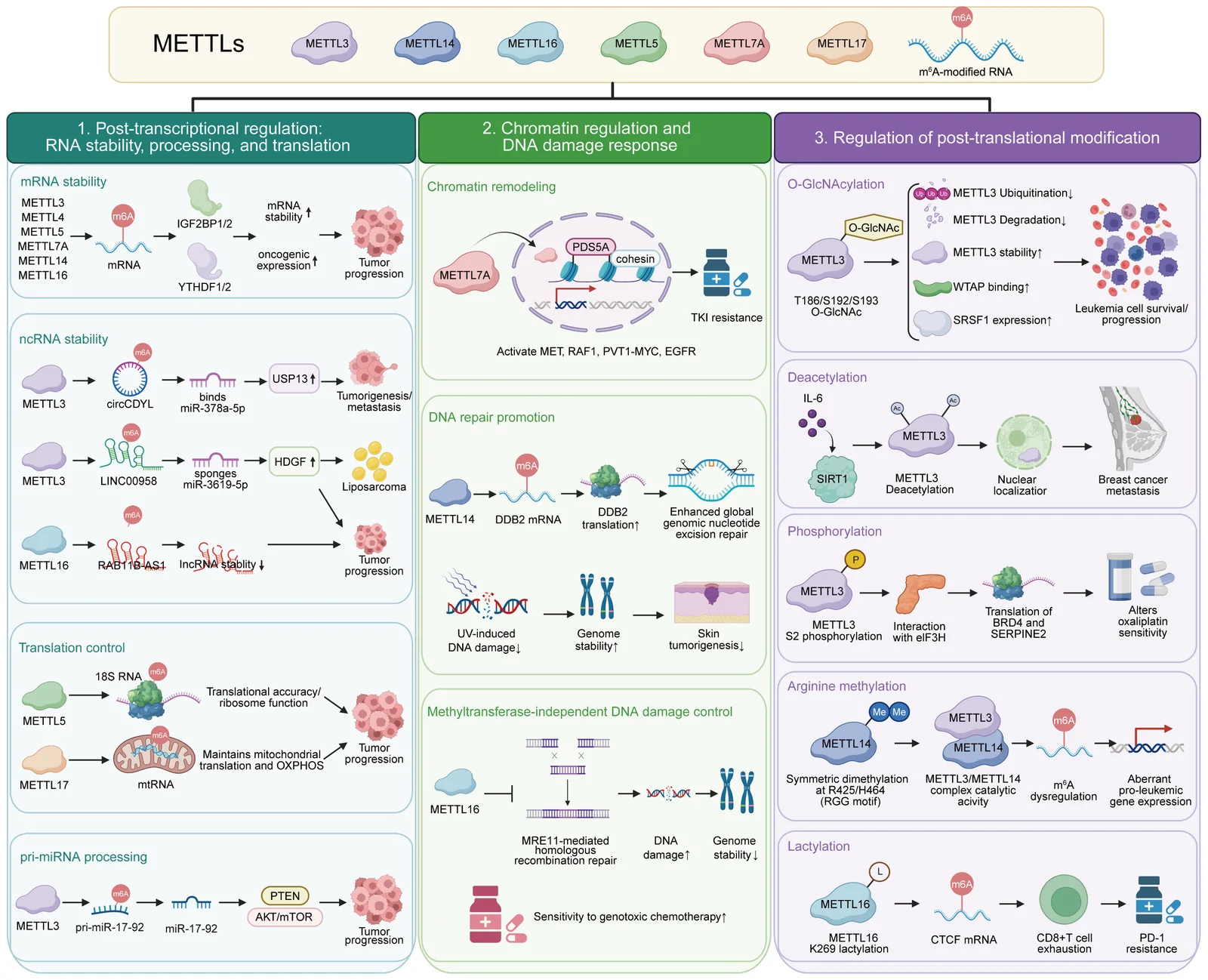
Schematic summary of regulatory roles of a subset of METTL family members in tumors. By controlling m6A-dependent and m6A-independent post-transcriptional processes, chromatin remodeling, DNA damage response, and post-translational modification, METTL members modulate tumorigenesis, metastasis, and drug resistance.

### METTL family proteins regulate RNA stability

Current experimental evidence indicates that RNA stability regulation mediated by classical m6A writers typically relies on m6A catalytic activity (**Table 2**). In cases where this has been validated, these proteins utilize their methyltransferase activity to catalyze m6A modifications on substrate RNA, which are subsequently recognized by specific m6A reader proteins that enact bidirectional biological effects on RNA stability or degradation. Importantly, variations in m6A abundance alone do not conclusively demonstrate a catalysis-dependent phenotype. Rescue experiments with catalytic-inactive mutants are considered the gold standard for differentiating between m6A catalytic-dependent functions and non-catalytic functions mediated by protein-protein interactions.

**Table 2:** Functional dependency classification of a subset of METTL family members and experimental evidence of their biological phenotypes.

METTL member	Biological phenotypes	Function-dependent classification	Regulatory roles in tumors	Experimental evidence supporting that classification	References
METTL3	METTL3 promotes gastric cancer progression	m6A-dependent (catalytic methyltransferase activity)	regulation of RNA stability	METTL3 knockdown reduces m6A level of STAT5A mRNA and downregulates STAT5A expression. The catalytic-inactive METTL3 mutant cannot restore STAT5A expression.	(55)
METTL3	METTL3 promotes the progression of gastric cancer	m6A-dependent (catalytic methyltransferase activity)	regulation of RNA stability	MeRIP-seq and MeRIP-qPCR verify m6A modification on circCDYL. Wild-type METTL3 overexpression increases m6A enrichment and the circCDYL expression, while the catalytic-inactive METTL3 mutant fails to produce such effects.	(44)
METTL3	METTL3 promotes HCC progression	unconfirmed	regulation of RNA stability	METTL3 directly binds to LINC00958 RNA and protects it from degradation via protein-RNA interaction alone, independent of its m6A methyltransferase activity. METTL3 functions as a scaffold to recruit other RNA-binding or RNA-stabilizing proteins onto LINC00958 without relying on its own m6A-catalytic capacity. Through interactions with other proteins, METTL3 indirectly perturbs intracellular RNA degradation cascades and LINC00958 half-life instead of exerting direct modification on LINC00958. METTL3 knockdown disturbs nuclear-cytoplasmic protein partitioning, leading to indirect changes in LINC00958 localization and stability. METTL3 might indirectly regulate LINC00958 by acting on other RNA-modifying enzymes.	(45)
METTL3	METTL3 promotes HCC progression	m6A-dependent (catalytic methyltransferase activity)	regulation of RNA processing	Wild-type METTL3, but not the catalytic-inactive METTL3 mutant, rescues reduced pri-miR-93 m6A levels and restores miR-93-5p expression in METTL3 knockdown tumor cells.	(140)
METTL3	METTL3 promotes the progression of gastric cancer	m6A-dependent (catalytic methyltransferase activity)	regulation of RNA processing	METTL3 increases m6A enrichment on pri-miR-17–92 and strengthens the binding between DGCR8 and pri-miR-17-92. pri-miR-17–92 A879C point mutation abolishes m6A modification, impairs miRNA maturation and eliminates oncogenic phenotypes.	(141)
METTL3	METTL3 promotes the progression of esophageal squamous cell carcinoma	m6A-dependent (catalytic methyltransferase activity)	participation in translation regulation	WASF3 is identified as a direct m6A target of METTL3. The catalytic-inactive METTL3 mutant fails to upregulate WASF3 protein.	(143)
METTL3	METTL3 promotes the progression of gastric cancer	m6A-independent (subcellular localization; protein-protein interactions; RNA-binding activity)	participation in translation regulation	Most METTL3-bound transcripts lack m6A modification. Catalytic-inactive mutant loses cellular m6A-writing capacity but still rescues proliferation, migration, and *in vivo* tumor-promoting phenotypes. Both the NLS-mutant METTL3-cyto (retained in the cytoplasm) and METTL3-mut-cyto (cytoplasmic location with inactivated enzyme activity) promote tumor malignant phenotypes. Re-expression of wild-type METTL3 or METTL3-cyto rescues tumor phenotypes in METTL3-knockout cells. Cytoplasmic METTL3 directly interacts with PABPC1 to form protein complexes, while this interaction is not disrupted after RNase A treatment. METTL3 binds target mRNAs with its own RNA-binding activity and facilitates translation via the PABPC1-eIF4F complex, independent of m6A modification for target recognition.	(50)
METTL3	O-GlcNAcylation of METTL3 promotes HCC progression	m6A-dependent (catalytic methyltransferase activity)	regulation of post-translational modification	METTL3 O-GlcNAc-deficient mutant cannot rescue the phenotype caused by METTL3 knockdown, accompanied by reduced m6A levels on MCM10 mRNA, weakened binding of IGF2BP3, and shortened mRNA half-life.	(147)
METTL3	O-GlcNAcylation of METTL3 promotes the progression of myelodysplastic syndrome and acute myeloid leukemia	m6A-dependent (catalytic methyltransferase activity)	regulation of post-translational modification	METTL3 O-GlcNAc-deficient mutants exhibit reduced m6A modification on SRSF1 mRNA and weakened binding to IGF2BP2.	(148)
METTL3	Nuclear METTL3 promotes breast cancer metastasis; cytoplasmic acetyl-mimetic METTL3 compromises metastatic capacity	m6A-dependent (catalytic methyltransferase activity); m6A-independent ( protein-protein interactions; subcellular localization)	regulation of post-translational modification	Re-expression of METTL3 wild-type or K177R (non-acetylatable) rescues tumor metastasis, but acetylation-mimetic K177Q mutant fails to rescue. K177Q retains in cytoplasm, reduces nuclear m6A levels and alters m6A levels on metastasis-related transcripts. Cytosolic K177Q enhances polysome formation and protein synthesis independent of METTL3 catalytic-inactive mutation. K177Q binds to the translation initiation complex eIF3h/eIF3b/CBP80 via protein interactions and promotes the translation of target mRNAs, independent of m6A modification. Acetylation at K177 cannot alter METTL3’s catalytic activity, but modulates subcellular distribution.	(149)
METTL3	S2 phosphorylation of METTL3 promotes resistance of gastric cancer to oxaliplatin	m6A-independent (Protein-protein interactions; RNA-binding activity)	regulation of post-translational modification	METTL3 S2A dephosphorylation mutant weakens the METTL3-eIF3H interaction without altering total m6A levels or the METTL3-METTL14 binding. This event triggers a decrease in BRD4 and SERPINE2 protein levels while mRNA abundance remains unchanged. METTL3 S2A dephosphorylation results in substantial attenuation of the interaction between METTL3 and eIF3H. METTL3 binds to target mRNAs BRD4 and SERPINE2 through its RNA-binding domain to recruit eIF3H to target transcripts.	(150)
METTL3	METTL3 promotes anti-PD-1 resistance	m6A-dependent (catalytic methyltransferase activity)	regulation of immune response	Overexpression of wild-type METTL3 upregulates CXCL1/5/CCL20 and downregulates PD-L1, whereas overexpression of the catalytic-inactive METTL3 mutant completely abolishes this effect.	(59)
METTL3	METTL3 promotes the progression of NAFLD-related HCC	m6A-dependent (catalytic methyltransferase activity)	regulation of immune response	METTL3 exerts no effect on SCAP mRNA levels but markedly augments SCAP translational efficiency. Overexpressing catalytic-inactive METTL3 mutant fails to upregulate SCAP protein.	(61)
METTL3	METTL3 promotes kidney cell cancer progression and immune evasion	m6A-dependent (catalytic methyltransferase activity)	regulation of immune response	Wild-type METTL3 restores PI3K and AKT phosphorylation in silenced cells. The catalytic-inactive D395A mutant cannot rescue this pathway activity, demonstrating that METTL3 regulation of PI3K/AKT depends on its m6A methyltransferase activity.	(40)
METTL3	METTL3 triggers PD-1 resistance and immunosuppression	m6A-dependent (catalytic methyltransferase activity)	regulation of immune response	METTL3 knockdown elevates PD-L1 and diminishes protein levels of CXCL1, CXCL5 and CCL20. Overexpression of wild-type METTL3, but not the catalytic-inactive METTL3 mutant downregulates PD-L1 and upregulates CXCL1, CXCL5 and CCL20.	(60)
METTL3	METTL3 promotes HCC progression	unconfirmed	regulation of immune response	METTL3 knockdown reduces m6A methylation of RBM14 mRNA and suppresses RBM14 mRNA and protein levels. YTHDF1 binds to RBM14 mRNA, and mutation of RBM14 m6A sites prevents such binding. METTL3 recruits YTHDF1 to bind to m6A sites on RBM14 and sustains RBM14 expression, depletion of YTHDF1 results in RBM14 downregulation.	(69)
METTL3	METTL3 promotes HCC progression	unconfirmed	regulation of immune response	m6A modification catalyzed by METTL3 stabilizes the lncRNA ZNNT1 and activates ZNNT1/OPN/S100A9 positive feedback crosstalk between HCC cells and macrophages. However, full functional rescue fails to occur for the catalytic-inactive METTL3 mutant and m6A site-mutated ZNNT1.	(71)
METTL3	METTL3 inhibits M2 polarization	unconfirmed	regulation of immune response	Changes in total m6A levels are detected upon METTL3 manipulation. However, key evidence from target-site validation and catalytically inactive mutant assays remains lacking, and non-catalytic functions as well as indirect secondary effects cannot be ruled out.	(154)
METTL3	METTL3 promotes glycolysis and proliferation of non-small cell lung cancer	unconfirmed	regulation of immune response	Changes in METTL3 expression are accompanied by alterations in NASP m6A modification and tumor malignant phenotypes. However, evidence for METTL3 catalytic-inactive mutants and NASP m6A site mutants remains unavailable. Thus, m6A-independent functions of METTL3 cannot be excluded.	(155)
METTL3	METTL3 promotes the NK cell-mediated antitumor immunity	unconfirmed	regulation of immune response	METTL3 knockout abolishes Prf1 and Gzmb m6A peaks and reduces the mRNA half-life. Exogenous supplementation of the methyl-donor SAM restores m6A levels and rescues the expression of effector molecules as well as the cytolytic function of NK cells. However, the lack of experiments with catalytic-inactive METTL3 mutants precludes the exclusion of m6A-independent functions.	(72)
METTL4	METTL4 promotes the progression of colorectal cancer	unconfirmed	regulation of RNA stability	METTL4 mediates TFRC mRNA m6A modification and promotes IGF2BP2 binding to TFRC mRNA. METTL4 knockdown decreases TFRC mRNA m6A levels as well as IGF2BP2-TFRC mRNA binding capacity. However, due to the lack of experiments with catalytic-inactive METTL4 mutants and m6A site mutations, the m6A-independent functions of METTL4 cannot be excluded.	(75)
METTL4	METTL4 promotes HCC progression	m6A-independent (Regulation of another RNA modification)	participation in translation regulation	The total cellular m6Am levels are reduced upon METTL4 knockdown, while m6A levels remain unaffected. METTL4-catalyzed m6Am modification elevates GLI1 mRNA translational efficiency to drive tumor proliferation and metastasis. Using catalytic-inactive METTL4 mutant and m6Am site-mutated GLI1 reporter, it can be proved that the oncogenic phenotype is dependent on METTL4’s m6Am catalytic activity (m6A-independent).	(36)
METTL5	METTL5 promotes the progression of osteosarcoma	unconfirmed	regulation of RNA stability	METTL5 overexpression leads to an increase in m6A modification on UBE3C mRNA and the extension of its half-life; METTL5 knockdown reduces both m6A modification and the half-life of UBE3C mRNA. However, knockdown or overexpression of METTL5 cannot strictly distinguish whether the phenotype arises from METTL5’s m6A catalytic activity or from its non-catalytic RNA binding and protein-protein interaction effects.	(81)
METTL5	METTL5 promotes HCC progression	m6A-dependent (catalytic methyltransferase activity)	participation in translation regulation	METTL5 catalytic-inactive mutant loses the capacity to elevate MGST1 protein, while MGST1 mRNA levels and mRNA half-life remain unchanged upon METTL5 knockdown.	(85)
METTL5	METTL5 promotes the progression of ovarian cancer	unconfirmed	participation in translation regulation	METTL5 remodels ribosomal scanning and selectively regulates ATF4 translation by modifying 18S rRNA rather than mRNA. Although omics analyses and downstream genetic rescue experiments provide solid evidence for its rRNA m6A-dependent mechanism, m6A-independent effects of METTL5 cannot be ruled out due to the lack of experiments with catalytic-inactive METTL5 mutants.	(82)
METTL5	METTL5 promotes the progression of lung squamous cell carcinoma	m6A-dependent (catalytic methyltransferase activity)	participation in translation regulation	METTL5 depletion specifically decreases m6A level of 18S rRNA, whereas total mRNA-m6A and genomic DNA m6dA modification are unaffected. Wild-type METTL5 rescues DEPDC1 protein expression, but catalytic-inactive mutant fails to restore the DEPDC1 protein levels.	(79)
METTL5	METTL5 promotes myeloma progression	unconfirmed	participation in translation regulation	Deletion or overexpression of METTL5 alters m6A levels of 18S rRNA A1832 and m6A-regulated SEPHS2 translation, but does not affect its transcription. Although omics analyses and downstream genetic rescue experiments provide evidence for its rRNA m6A-dependent mechanism, m6A-independent effects of METTL5 cannot be ruled out due to the lack of experiments with catalytic-inactive METTL5 mutants.	(146)
METTL5	METTL5 promotes immune escape in intrahepatic cholangiocarcinoma	m6A-dependent (catalytic methyltransferase activity)	regulation of immune response	Overexpression of wild-type METTL5 suppresses CXCL16 translation, whereas overexpression of the catalytic-inactive METTL5 mutant fails to suppress CXCL16 translation.	(11)
METTL7A	METTL7A promotes resistance of adenocarcinoma to tyrosine kinase inhibitors	m6A-independent (DNA-binding activity; Subcellular localization)	participation in chromatin regulation and DNA damage response	Under drug-tolerant persister state, METTL7A is transferred from the cytoplasm to the nucleus and directly binds to double-stranded DNA, while the K86A mutation at the key DNA-binding site prevents nuclear translocation and weakened DNA binding affinity.	(92)
METTL14	METTL14 suppresses HCC progression	m6A-dependent (catalytic methyltransferase activity)	regulation of RNA stability	MeRIP-qPCR verified m6A enrichment on the 5’UTR of SLC7A11 mRNA; the catalytically inactive METTL14-R298P mutant markedly reduces this m6A enrichment. In addition, the wild-type SLC7A11 5’UTR reporter is repressed upon METTL14 overexpression, while the m6A-site mutant reporter remains unresponsive to METTL14 overexpression.	(102)
METTL14	METTL14 suppresses ultraviolet-induced skin tumorigenesis	m6A-dependent (catalytic methyltransferase activity)	participation in chromatin regulation and DNA damage response	Knockdown of METTL14 reduces cellular m6A levels, DDB2 protein abundance, and cyclobutane pyrimidine dimer repair. Overexpression of wild-type METTL14, but not catalytic-inactive R298P mutant, rescues m6A level and restores genome-wide nucleotide excision repair capacity.	(96)
METTL14	Symmetric dimethylation of METTL14 promotes leukemia progression	m6A-dependent (catalytic methyltransferase activity)	regulation of post-translational modification	The catalytic efficiency of the METTL3/14 complex is substantially impaired by the demethylation-deficient mutant (R425/455K). Total mRNA levels of BRD4, SP1, and c-MYC are unchanged, yet m6A peaks on these transcripts are decreased, and aberrant protein expression cannot be rescued.	(153)
METTL14	METTL14 maintains the stability of Treg cells	unconfirmed	regulation of immune response	METTL14 catalyzes m6A modification on the 3’UTR of Sema4D mRNA, which is recognized by YTHDF2 to facilitate Sema4D transcript degradation. Loss of METTL14 abolishes 3’UTR m6A modification of Sema4D mRNA, augments the mRNA stability, and impairs Treg function. However, m6A-independent effects of METTL14 cannot be ruled out due to the lack of experiments with catalytic-inactive METTL14 mutants.	(107)
METTL14	METTL14 enhances glioblastoma progression and immune evasion	unconfirmed	regulation of immune response	METTL14 catalyzes m6A modification on PD-L1 mRNA, which is recognized by IGF2BP2 to enhance transcript stability and upregulate PD-L1 protein. However, m6A-independent effects of METTL14 cannot be excluded due to the lack of experiments with catalytic-inactive METTL14 mutants and m6A site-mutated PD-L1.	(108)
METTL16	METTL16 promotes the progression of colorectal cancer	m6A-dependent (catalytic methyltransferase activity)	regulation of RNA stability	METTL16 modulates m6A enrichment on TM7SF2 transcript. SRAMP prediction, RIP-MeRIP and wild-type/mutant dual luciferase reporter verified the functional m6A site within TM7SF2.	(116)
METTL16	METTL16 promotes the progression of colorectal cancer	m6A-dependent (catalytic methyltransferase activity)	regulation of RNA stability	METTL16 modulates m6A enrichment on MSMO1 transcript. The catalytic-inactive METTL16 mutant fails to restore MSMO1 expression and p38-MAPK phosphorylation.	(117)
METTL16	Phosphorylation of METTL16 promotes the progression of clear cell renal cell carcinoma	unconfirmed	regulation of post-translational modification	CDK13 directly binds to and phosphorylates METTL16 Ser329 to enhance the m6A catalytic function of METTL16. METTL16 catalyzes 3’UTR m6A modification on ACLY mRNA, which is recognized by YTHDC2 to enhance ACLY mRNA stability and upregulate ACLY protein. Although multiple lines of indirect evidence support an m6A-dependent mechanism, m6A-independent effects of METTL16 cannot be excluded due to the lack of experiments with catalytic-inactive METTL16 mutants and m6A site-mutated ACLY.	(151)
METTL16	METTL16 promotes HCC progression	unconfirmed	regulation of RNA stability	METTL16 directly binds to lncRNA RAB11B-AS1 and reduces the transcript stability of RAB11B-AS1 via m6A modification to accelerate tumor progression. RAB11B-AS1 overexpression rescues METTL16-mediated tumor phenotypes. However, m6A-independent effects of METTL16 cannot be excluded due to the lack of experiments with catalytic-inactive METTL16 mutants and m6A site-mutated RAB11B-AS1.	(112)
METTL16	METTL16 promotes chemoresistance in TNBC	m6A-dependent (catalytic methyltransferase activity)	participation in translation regulation	Catalytic-inactive mutant METTL16-N184A loses *in vitro* methyltransferase activity and fails to induce taxane-resistance phenotype. Wild-type METTL16 transfers ABCB1 mRNA to polysome fractions to enhance translation efficiency; N184A mutant cannot enhance ribosome loading.	(118)
METTL16	METTL16 promotes the progression of myeloma	m6A-independent (Protein-protein interactions)	participation in translation regulation	Independent of methyltransferase activity, METTL16 directly interacts with PERK and eIF2α via its MTase domain, competitively disrupting the PERK-eIF2α complex to block eIF2α phosphorylation and reverse mRNA translational inhibition. METTL16 augments Cyclin D1 protein translation, and overexpression of the catalytic-inactive METTL16 mutant is still able to upregulate Cyclin D1 protein.	(29)
METTL16	METTL16 induces increased sensitivity of tumor cells to genotoxic chemotherapeutic agents	m6A-independent (RNA-binding activity)	participation in chromatin regulation and DNA damage response	METTL16 suppresses MRE11 exonuclease activity and blocks DNA end resection by forming an RNA-dependent METTL16-RNA-MRE11complex. Catalytic-inactive METTL16-F187G mutant still interacts with MRE11 and retains homologous recombination inhibitory function. METTL16 binds to the MRE11-RAD50-NBS1 complex, and RNase treatment but not DNase disrupts this interaction.	(119)
METTL16	Lactylation of METTL16 suppresses CD8+ T cell cytotoxicity and induced PD-1 immunotherapy resistance	unconfirmed	regulation of post-translational modification	METTL16 K269 lactylation enhances m6A catalytic activity, increases CTCF mRNA stability and protein levels, and consequently activates transcription of immunosuppressive molecules. Although multiple evidence supports an m6A-dependent mechanism, m6A-independent effects of METTL16 cannot be excluded due to the lack of experiments with catalytic-inactive METTL16 mutants.	(152)
METTL16	METTL16 promotes HCC progression	unconfirmed	regulation of immune response	m6A modification catalyzed by METTL16 stabilizes the lncRNA ZNNT1 and activates ZNNT1/OPN/S100A9 positive feedback crosstalk between HCC cells and macrophages. However, full functional rescue fails to occur for the catalytic-inactive METTL16 mutant and m6A site-mutated ZNNT1.	(71)
METTL17	METTL17 promotes the progression of colorectal cancer	unconfirmed	participation in translation regulation	METTL17 regulates multiple mtRNA methylations (i.e., m6A, m4C, m1C, m1G) and interactes with mitochondrial ribosomal small subunit proteins to maintain the synthesis of mitochondrial encoded proteins and oxidative phosphorylation. However, m6A-independent effects of METTL17 cannot be excluded due to the lack of experiments with catalytic-inactive METTL17 mutants.	(31)
METTL17	METTL17 promotes the progression of acute myeloid leukemia	m6A-independent (Regulation of another RNA modification; Subcellular localization)	participation in translation regulation	METTL17, via mitochondrial localization and its methyltransferase activity, catalyzes methylation of mt-12S rRNA at the m4C839 and m5C841 sites, not m6A, to sustain 12S rRNA stability, mitochondrial translation and oxidative phosphorylation.	(122)

In the context of mRNA stability regulation, the recognition of m6A sites by IGF2BP1/2 or YTHDF1 offers protection against degradation (55–57, 75, 138). For instance, METTL3 catalyzes m6A modification throughout the coding region of STAT5A mRNA, leading to the recruitment of IGF2BP2, which stabilizes the transcript and promotes tumor growth and metastasis (55). Similarly, METTL3 catalyzes the m6A modification of NDUFA4 mRNA, which recruits IGF2BP1 to inhibit degradation and thereby facilitate tumor progression (56, 57). The loss of METTL4 has been shown to alter the m6A abundance of TFRC mRNA, with IGF2BP2 directly binding to TFRC mRNA to maintain its stability, thus promoting tumor growth (75). However, the catalytic dependency of this phenotype on m6A remains unverified, as no rescue assay using catalytic-inactive mutants has been conducted, leaving the possibility of non-catalytic functions open. METTL16 enhances TM7SF2 mRNA stability via the m6A-IGF2BP1/2 axis (116) or and stabilizes MSMO1 mRNA through the m6A-IGF2BP2 axis, contributing to tumorigenesis (117). By facilitating m6A modification to improve UBE3C mRNA stability, METTL5 enhances osteosarcoma growth and metastasis (81). While this oncogenic effect is likely m6A-dependent, it remains uncertain whether METTL5 executes this function through protein-protein interactions instead of m6A methylation.

The METTL3/14 complex and METTL14 demonstrate bidirectional regulatory effects on RNA stability, with this bidirectionality emanating not from these proteins themselves, but rather from downstream m6A readers (139). Recognition of the m6A site by proteins such as YTHDF2 or YTHDC2 activates RNA degradation processes, which depend on the m6A catalytic activity. For instance, the METTL3/14 complex catalyzes m6A modification in a manner dependent on its methyltransferase activity. Activation of the YTHDC2/YTHDF2 axis results in reduced stability of MRP7 mRNA, thereby reversing chemoresistance (58). Under hypoxic conditions, m6A modification mediated by METTL14 induces the degradation of SLC7A11 mRNA via the m6A-YTHDF2 pathway, ultimately leading to tumor ferroptosis (102).

While mRNA is primarily addressed, ncRNAs can also be influenced by m6A writers; however, strict catalytic dependence on m6A has not been validated for some regulatory mechanisms involving ncRNAs. Recent studies indicate that METTL3 modifies circCDYL through an m6A-dependent process, which enhances circCDYL’s binding affinity for miR-378a-5p, resulting in the upregulation of USP13 and driving tumorigenesis and metastasis (44). For the lncRNA LINC00958, it has been observed that knockdown of METTL3 reduces the m6A modification levels of LINC00958 transcripts, consequently shortening their RNA half-life, downregulating LINC00958, and inhibiting lipogenesis as well as tumor progression in liver cancer (45, 46). Although correlations between METTL3, m6A modification, LINC00958 stability, and tumor phenotypes have been established, the absence of experiments involving a catalytic-inactive mutant prevents the complete exclusion of METTL3’s non-catalytic roles in this biological process. In contrast, overexpression of METTL16 has been shown to shorten the half-life of lncRNA RAB11B-AS1 transcripts, corresponding with increased m6A modification levels (112), thereby suggesting a potential m6A-dependent mechanism for lncRNA degradation. However, as no experiments with catalytically inactive METTL16 mutants were conducted, the possible involvement of METTL16’s m6A non-catalytic functions cannot be entirely ruled out.

### METTL family proteins regulate RNA processing

Moreover, METTLs play a significant role in RNA processing and maturation, particularly in the splicing and maturation of primary microRNAs (pri-miRNAs). Two experimentally validated METTL regulatory mechanisms, which both rely on m6A catalysis, are outlined below (**Table 2**).

Research demonstrates that m6A methylation on pri-miR-93, catalyzed by METTL3, enhances the expression of miR-93-5p, thus accelerating cell cycle progression in tumor cells by inhibiting CDKN1A (140). Rescue assays confirmed that wild-type METTL3 could restore m6A levels of pri-miR-93 and reconstitute the production of mature miR-93-5p in tumor cells with METTL3 knockdown, whereas the catalytic-inactive METTL3 mutant failed to achieve this (140). Similarly, m6A methylation on pri-miR-17-92, catalyzed by METTL3, promotes its processing into matured miR-17-92, which subsequently activates the AKT/mTOR oncogenic pathway by targeting tumor suppressor genes such as PTEN (141). The mutation experiment at the pri-miR-17–92 A879C site further confirms this m6A-dependent mechanism (141).

### METTL family proteins participate in translation regulation

METTL family proteins regulate protein synthesis efficiency at the translational level through both m6A catalytic-dependent and -independent mechanisms, collectively remodeling tumor phenotypes (**Table 2**). Early investigations have primarily produced correlational outcomes through gene knockout strategies, lacking experimental validation with catalytic-inactive mutants; therefore, non-catalytic effects cannot be entirely discounted.

In m6A-dependent regulatory pathways, METTL3 enhances the translation efficiency of oncogenes such as SPHK2, ENO1, SLC7A11, and WASF3 through the m6A-YTHDF1 or m6A-IGF2BP2 axes, thereby accelerating tumor progression (142, 143). METTL5 increases MGST1 expression at the translational level via its m6A catalytic activity, granting ferroptosis resistance to tumor cells (85). Additionally, METTL16 improves the translation efficiency of ABCB1 in an m6A-dependent manner, driving paclitaxel resistance in triple-negative breast cancer (TNBC) (118).

In ovarian cancer models, METTL5 deficiency specifically decreases m6A modification levels of 18S rRNA, without affecting m6A abundance in mRNA or 28S rRNA (82). These molecular changes correspond with impaired ATF4 translation efficiency and inhibition of SLC7A11 and SLC3A2, making tumor cells more susceptible to T cell-mediated ferroptosis (82). Evidence also suggests that METTL5-mediated m6A modification of 18S rRNA influences the translation of oncogenes such as DEPDC1 (79). Furthermore, METTL5 deficiency disrupts ribosomal translation fidelity, activates the translation of unannotated open reading frames, generates tumor immunogenic neoantigens, reshapes the tumor immune microenvironment, and enhances antitumor immune responses (144, 145). In multiple myeloma, METTL5 knockout results in decreased m6A at site A1832 of 18S rRNA, selectively enhancing SEPHS2 translation and preserving selenoprotein synthesis and cellular redox homeostasis (146). All described METTL5-related phenotypes correlate with alterations in 18S rRNA m6A modification; however, without catalytic inactivation mutation experiments, the non-catalytic functions of METTL5 cannot be ruled out.

In m6A-independent regulatory pathways, METTL3 directly interacts with the translation-related factor PABPC1 to facilitate the translation of specific epigenetic factors, thereby promoting tumor progression (50). METTL16 enhances the translation of PSMB5 and CCND1 through the PERK-eIF2α pathway, independent of its m6A modification, contributing to resistance against proteasome inhibitors (29). By catalyzing N6, 2’-O-dimethyladenosine (m6Am) modification, METTL4 enhances GLI1 translation efficiency and promotes tumorigenesis (36).

Mitochondrial-localized METTL17 exhibits a unique pattern of translational regulation. Unlike canonical m6A-related METTLs, METTL17 uses mtRNA as a direct catalytic substrate to perform multiple RNA methylations, including m6A, m4C, m7G, m3C, and m5C (31). By integrating diverse mtRNA modifications, it regulates mitochondrial gene translation and oxidative phosphorylation (OXPHOS), further influencing tumor progression (31). Recent studies indicate that inhibiting METTL17 can obstruct mitochondrial translation, suppress OXPHOS, and ultimately inhibit colorectal cancer progression (31). It is important to note that m6A methylation is not a universal function for all METTL family proteins, and METTL17 is a representative non-canonical member. Most published studies have not specifically linked METTL17’s oncogenic phenotype to m6A modification. Several investigations lack catalytic-inactive mutant rescue assays, making it difficult to distinguish its RNA methyltransferase activity from its role as a Fe-S cluster-dependent ribosome assembly scaffold. Other studies have confirmed that METTL17-mediated non-m6A modifications (such as m4C and m5C) sustain mitochondrial translation, OXPHOS, and leukemia stem cell survival, demonstrating strict dependence on its catalytic activity (122). Collectively, these findings suggest that METTL17 modulates tumor energy metabolism and malignant progression through multiple mtRNA methylation modifications, indicating that METTLs exhibit significant functional diversity while highlighting that m6A modification cannot universally explain tumor regulatory mechanisms across all METTLs.

### METTL family proteins participate in chromatin regulation and DNA damage response

METTL7A is categorized as a non-classical METTL member and primarily exerts chromatin regulatory functions through an m6A-independent mechanism (92). Historically, METTL7A was thought to localize in the endoplasmic reticulum and nucleus without binding to chromatin. However, recent findings have revised this perspective. During the transition phase of EGFR-mutant lung adenocarcinoma developing resistance to tyrosine kinase inhibitors (TKIs), METTL7A translocates to the nucleus. The TKI resistance phenotype mediated by METTL7A is independent of m6A and relies on its double-stranded DNA binding activity and nuclear chromatin localization. This process facilitates the recruitment of PDS5A to the chromatin through protein-protein interactions, regulating cohesin loading and genome remodeling, ultimately activating chromatin states of oncogenic loci such as MET, RAF1, PVT1-MYC, and EGFR, thereby inducing TKI resistance (92). This study presents a model illustrating how RNA-modifying proteins can regulate genome stability through non-catalytic DNA-binding activity.

In DNA damage responses, the biological effects mediated by METTL14 are m6A-dependent (96, 97). METTL14 directly targets DDB2 mRNA as its substrate and catalyzes m6A modification, promoting DDB2 translation, enhancing genome-wide nucleotide excision repair capacity, reducing ultraviolet-induced carcinogenic DNA damage, maintaining genomic stability, and restraining skin tumorigenesis (96, 97). Conversely, METTL16 suppresses homologous recombination repair in an m6A-independent manner (42, 119, 120). It does not directly modify DNA but represses MRE11-driven homologous recombination repair through protein-protein interactions, leading to the accumulation of DNA damage, genomic instability, and increased sensitivity of tumor cells to genotoxic chemotherapeutic agents (42, 119, 120).

### METTL family proteins regulate post-translational modification

METTLs undergo regulation by various post-translational modifications, including O-GlcNAcylation, deacetylation, phosphorylation, lactylation, and arginine methylation (**Figure 2**). Notably, the regulation of METTLs through post-translational modification exhibits clear m6A-dependent and m6A-independent dual modes (**Table 2**).

O-GlcNAcylation of METTL3 is recognized as a frequent occurrence in hepatocellular carcinoma (HCC) and myeloid hematologic malignancies. O-GlcNAcylation at specific sites (T186/S192/S193) can inhibit METTL3 ubiquitination and degradation, thereby enhancing its binding to the cofactor protein WTAP and promoting HCC progression (147). This carcinogenic phenotype is confirmed to depend on the m6A catalytic activity of METTL3. Additionally, O-GlcNAcylation increases METTL3 stability and upregulates SRSF1 mRNA via an m6A-dependent catalytic mechanism, thereby enhancing leukemic cell survival (148).

METTL3 acetylation is a major determinant of its subcellular localization and biological functions. Through SIRT1-dependent deacetylation, the inflammatory factor IL-6 stimulates METTL3 nuclear translocation, contributing to breast cancer metastasis (149). This process reveals dual modes of regulation, as reduced nuclear m6A accumulation correlates with a metastasis-suppressive phenotype, while the cytoplasmic acetyl-mimetic METTL3 mutant (K177Q) promotes polysome formation and protein synthesis, independent of METTL3 catalytic inactivation (149).

Phosphorylation of METTL3 at the S2 site regulates its interaction with the translation initiation factor eIF3H, which subsequently impacts the translational activity of BRD4 and SERPINE2, thereby reshaping tumor cell sensitivity to oxaliplatin (150). The S2A dephosphorylation mutant weakens the METTL3-eIF3H interaction without affecting total m6A levels or the METTL3-METTL14 binding (150). This alteration triggers a decrease in BRD4 and SERPINE2 protein levels, while mRNA abundance remains unchanged, indicating that the mechanism operates independently of m6A catalysis (150). In contrast, increasing evidence suggests that phosphorylation of METTL16 at S329 by CDK13 accelerates the progression of clear cell renal cell carcinoma through m6A modification of ACLY mRNA in an m6A-dependent manner; however, confirmation of its m6A catalytic activity awaits further mutation experiments (151).

Lactate uptake by Schwann cells induces lactylation of METTL16 at K269, enhancing its m6A methyltransferase activity. This modification is accompanied by stabilized CTCF mRNA and elevated expression of CD276 and NECTIN2, ultimately suppressing CD8+ T cell cytotoxicity (152). Although this study supports an m6A-dependent cascade, it cannot completely rule out contributions from catalytic-independent functions of METTL16.

Symmetric dimethylation of METTL14 at R425 and R445 within the RGG motif enhances the catalytic activity of the METTL3/14 complex, increases m6A modification on target mRNAs, and elevates the expression of key pro-leukemic genes (153). This regulatory mechanism is therefore dependent on m6A catalysis.

## Regulatory mechanisms of METTL family proteins in tumor immune response

A systematic summary of the regulatory roles of core METTL family members in tumor formation and development highlights that, beyond the inherent aberrant proliferation of tumor cells, tumor progression is closely tied to immune dysregulation within the TME. Growing evidence indicates that these METTLs exert bidirectional and context-dependent immunoregulatory effects within the TME (**Table 3**), with their pro-tumor or antitumor immune influences dependent on intrinsic cellular functions (such as those of tumor cells, T cells, macrophages, and natural killer (NK) cells), tumor type, and extrinsic signals from the TME (including hypoxia, cytokines, and metabolic reprogramming). This section provides a comprehensive elaboration on the bidirectional immunoregulatory functions of these proteins and analyzes the upstream signals and downstream immune cascade networks that contribute to tumor heterogeneity (**Figure 3**).

**Table 3:** Summary of direct and indirect immunoregulatory mechanisms of a subset of METTL family members in the TME.

METTL member	Cell types directly regulated by METTLs	Target RNA	Reader/pathway	Immune phenotype	Experimental model	Reference
Tumor cell-intrinsic METTL effects						
METTL3	tumor cells	BHLHE41 mRNA	m6A-BHLHE41-CXCL1/CXCR2	reduced CD8+ T cell infiltration and activation	METTL3 conditional knockout mice; METTL3 knockdown tumor cells	(59)
METTL3	tumor cells	c-Myc mRNA	m6A-c-Myc-CXCL1/CXCL5/CCL20; METTL3-m6A-PD-L1	reduced CD8+ T and NK cell infiltration	METTL3 conditional knockout mice; METTL3 knockdown tumor cells	(60)
METTL3	tumor cells	SCAP mRNA	m6A-SCAP-SREBP2	reduced CD8+ T cell effector function	Hepatocyte-specific METTL3 knock-in/heterozygous knockout mice	(61)
METTL3	tumor cells	SLC16A1-AS1 lncRNA	m6A-SLC16A1-AS1-HNRNPA1-SLC16A1	promotion of M2 TAM polarization	Mice with subcutaneous xenograft tumors	(167)
METTL3	tumor cells	PD-L1 mRNA	YTHDF1	increased PD-L1 expression; reduced CD8+ T cell infiltration and cytotoxicity	Mice with subcutaneous xenograft tumors	(40)
METTL3	tumor cells	PD-L1 mRNA	IGF2BP3	increased PD-L1 expression; T cell exhaustion	Mice with subcutaneous xenograft tumors	(65)
METTL3	tumor cells	PD-L1 mRNA	IGF2BP1	increased PD-L1 expression; tumor immune escape	Mice with subcutaneous xenograft tumors	(66)
METTL3	tumor cells	MALAT1 lncRNA	m6A-MALAT1-PD-L1	increased PD-L1 expression	Human PC cells with gene knockdown/overexpression	(67)
METTL3	tumor cells	PD-L1 mRNA	YTHDF2	decreased PD-L1 expression; reduced CD8+ T cell infiltration	Mice with subcutaneous xenograft tumors	(68)
METTL3/METTL16	tumor cells	ZNNT1 lncRNA	m6A-ZNNT1-OPN-S100A9	promotion of M2 TAM polarization	ZNNT1 knockdown/overexpressed SNU-398 cells subcutaneous in Balb/c nude mice; ZNNT1 knockdown/overexpressed tumor cells	(71)
METTL5	tumor cells	18S rRNA	m6A-18S rRNA-CXCL16/CX3CL1	reduced CD8+ T cell infiltration; promotion of M2 TAM polarization	Hepatocyte-specific METTL5 conditional knockout mice; METTL5-knockout tumor cells	(11)
METTL5	tumor cells	COPS6 mRNA	YTHDF1	reduced CD8+ T cell infiltration and cytotoxicity; elevated PD-L1 expression	Balb/c mice model with *in vivo* rescue (METTL5 overexpression); METTL5 knockdown tumor cells	(86)
METTL5	tumor cells	18S rRNA	m6A-18S rRNA-ATF4-SLC7A11/SLC3A2	reduced CD8+ T cell cytotoxicity	METTL5 knockout ID8/GC cells subcutaneous in NSG and C57BL/6 mice; METTL5 knockout tumor cells	(82)
METTL7B	tumor cells	PD-L1 mRNA	JAK1/2-STAT3-PD-L1	decreased PD-L1 expression	METTL7B knockdown/overexpressed tumor cells	(93)
METTL14	tumor cells	PD-L1 mRNA	IGF2BP2	increased PD-L1 expression; reduced T cell cytotoxicity; T cell exhaustion	METTL14 knockdown U251 cells subcutaneous in Balb/c nude mice; METTL14 knockdown tumor cells	(108)
METTL14	tumor cells	AMPKα mRNA	m6A-AMPKα-GPR81-PD-1	promotion of M2/TAM polarization; increased PD-1 expression in macrophages	METTL14 knockdown TC-1 cells subcutaneous in C57BL/6 mice; METTL14 knockdown/overexpressed tumor cells	(111)
METTL16	tumor cells	PD-L1 mRNA	IGF2BP2	increased PD-L1 expression; tumor immune escape	Mice with subcutaneous xenograft tumors	(173)
Immune cell-intrinsic METTL effects						
METTL3	Treg cells	Socs family mRNAs	m6A-Socs/Cish-IL-2-STAT5	enhanced Treg suppressive function	Treg-specific METTL3 knockout mice	(63)
METTL3	Th17 cells	Socs3 mRNA	m6A-SOCS3-IL-6-STAT3-IL-17A/CCR5	increased Th17 cell differentiation	Th17-specific METTL3 knockout mice	(64)
METTL3	Tfh cells	TCF-1 mRNA	m6A-TCF-1-Bcl-6/CXCR5/ICOS	increased Tfh cell differentiation	CD4+ T cell-specific METTL3 knockout mice	(159)
METTL3	kupffer cells (liver macrophages)	RBM14 mRNA	YTHDF1	promotion of M2 macrophage polarization	METTL3 knockdown Kupffer cells subcutaneous in Balb/c nude mice	(69)
METTL3	bone marrow-derived macrophages	SPRED2 mRNA	YTHDF1	decreased M1/M2 macrophage mixed activation and Treg recruitment	Myeloid/macrophage-specific METTL13 knockout mice	(161)
METTL3	NK cells	SHP-2 mRNA	m6A-SHP-2-AKT-mTOR/MAPK-ERK	increased NK cell proliferation and maturation	NK cell-specific METTL3 knockout mice	(162)
METTL3	Human ESC-derived NK cells	–	T-BET/EOMES-PRF1/GZMB/IFNG	decreased NK cell production	METTL3 knockdown hESC cells, mature hESC-NK cells	(163)
METTL3	macrophages	OAS3 mRNA	m6A-OAS3-IL-10/VEGF-A	increased M2d macrophage infiltration; reduced CD8+ T cell cytotoxicity	Humanized PC mice	(103)
METTL3/14	NK cells	PRF1/GZMB mRNA	IGF2BP1/IGF2BP3	enhanced NK cell antitumor cytotoxicity	NK cell-specific METTL3/14 knockout mice	(72)
METTL14	Treg cells	Sema4D mRNA	YTHDF2	enhanced Treg suppressive function	Treg-specific METTL14 knockout mice	(107)
METTL14	CD8+ T cells	PD-1 mRNA	YTHDF1/2/3	reduced PD-1 expression; improved CD8+ T cell effector function	T cell-specific METTL14 knockout mice	(110)
METTL14	NK cells	SOCS3 mRNA	m6A-SOCS3-IL-15-JAK-STAT5	excessive activation of NK cells	NK progenitor-stage-specific METTL14 knockout mice; mature NK cell-stage-specific METTL14 knockout mice	(164)

**Figure 3:**
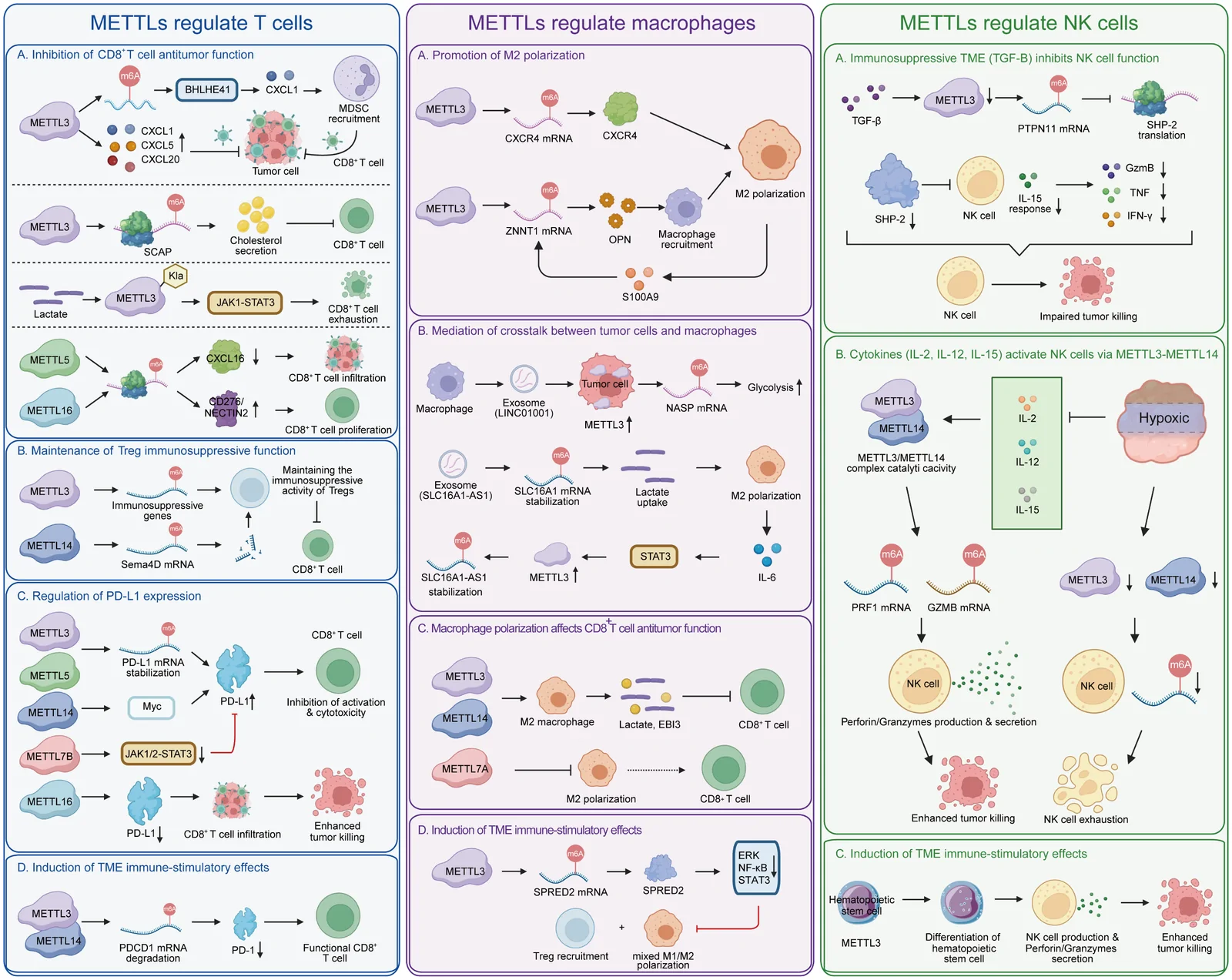
Regulatory mechanisms of a subset of METTL family members in tumor immune response.

### Tumor cell-intrinsic METTL functions

This section focuses on the intrinsic roles of METTLs in tumor cells, which remodel the TME by modulating various molecular mechanisms, including chemokine secretion, immune checkpoint expression, and the release of soluble mediators.

m6A writers in tumor cells regulate chemokine expression through m6A modification, influencing the recruitment and infiltration of immune cells. For instance, METTL3 activates the m6A-BHLHE41-CXCL1 axis, facilitating the recruitment of myeloid-derived suppressor cells to tumor tissues and indirectly weakening the effector functions of CD8+ T cells (59). By means of its m6A catalytic activity, tumor cell-intrinsic METTL3 stimulates the upregulation of chemokines such as CXCL1, CXCL5, and CCL20, thereby creating a non-inflammatory TME that hinders CD8+ T cell infiltration into tumor lesions (60). Specific blockade of METTL3’s m6A methyltransferase activity can reverse immunosuppression mediated by METTL3, activate tumor cell-intrinsic interferon signaling, remodel the TME to be pro-inflammatory, and significantly enhance antitumor immune responses (156). Additionally, intrinsic METTL5 in tumor cells suppresses CXCL16 secretion through m6A-dependent catalysis, obstructing CD8+ T cell infiltration and proliferation (11, 86).

Beyond regulating immune cell recruitment mediated by chemokines, METTL5 modulates the inherent sensitivity of tumor cells to T cell cytotoxicity through the reprogramming of rRNA translation. In ovarian cancer, tumor cell-intrinsic METTL5 mediates m6A methylation of 18S rRNA, which sustains ATF4 translation. This, in turn, transcriptionally activates SLC7A11 and SLC3A2, enhancing the antioxidant capacity of tumor cells, antagonizing CD8+ T cell-induced ferroptosis, and ultimately leading to resistance to immunotherapy (82). Targeting METTL5-mediated immune suppression presents an opportunity to improve the efficacy of antitumor therapies (157).

Programmed death-ligand 1 (PD-L1) on tumor cell membranes is a key factor in immune escape. Several METTLs in tumor cells can directly regulate PD-L1 expression post-transcriptionally. METTL3 utilizes its m6A methyltransferase activity to upregulate PD-L1 expression by stabilizing PD-L1 mRNA (40, 65–67). Similarly, METTL14 catalyzes the m6A modification of PD-L1 mRNA, which is recognized by IGF2BP2, leading to increased stability and protein levels of PD-L1 (108). In contrast, as an m6A writer, METTL16 significantly restrains PD-L1 expression in tumor cells (121). Other RNA methyltransferases, such as METTL7B, can attenuate the PD-L1-mediated immunosuppressive effects on T cells via the JAK-STAT3 pathway (93). Clinical sample analyses have demonstrated a close correlation between METTL3 and METTL16 expression and PD-L1 levels in tumor tissues (158), indicating that these tumor cell-intrinsic METTLs are critical upstream regulators of PD-L1 expression.

Tumor cells also transmit regulatory signals from m6A writers to immune cells through the secretion of soluble mediators and exosomes. In HCC, METTL3, along with METTL16, catalyzes m6A modification on the lncRNA ZNNT1, enhancing the stability of ZNNT1 transcripts. The upregulated ZNNT1 leads to increased secretion of osteopontin (OPN), promoting macrophage recruitment and M2 macrophage polarization (38, 71). Furthermore, macrophages can deliver lncRNA LINC01001 to tumor cells via exosomes (155). This lncRNA interacts with METTL3 to trigger m6A methylation modification of NASP, enhancing tumor glycolysis and proliferation, thus establishing a bidirectional regulatory loop between tumor cells and macrophages (155).

### T cell-intrinsic METTL functions

T cells are fundamental effector cells in adaptive antitumor immunity. METTL3 and METTL14, as core components of the m6A methyltransferase complex, play pivotal roles in directly regulating T cell differentiation, effector functions, exhaustion, and immune checkpoint expression.

#### Regulation of Tregs and Th cells

Regulatory T cells (Tregs) are key subsets of CD4+ T cells that drive tumor immunosuppression. Through cell-intrinsic mechanisms, METTL3 and METTL14 sustain the immunosuppressive functions of Tregs, which further dampen the antitumor effects of CD8+ T cells. Specifically, in Treg cells, METTL3 catalyzes m6A methylation on Socs family mRNAs, triggering their degradation. This alleviates repression of the IL-2-STAT5 signaling axis, thereby maintaining the suppressive properties of Tregs (63). Deletion of METTL3 specifically within Tregs induces Treg dysfunction and a loss of immunosuppressive activity (63). METTL14 directly regulates the differentiation of inducible Tregs (iTregs) and sustains FoxP3 expression via the mTOR signaling pathway (106). A deficiency in METTL14 disrupts iTreg differentiation, reduces FoxP3 expression, and impairs the suppressive capacity of iTregs both *in vitro* and *in vivo* (106). Moreover, m6A modification catalyzed by METTL14 facilitates YTHDF2-mediated degradation of Sema4D mRNA, contributing to the functional stability of Tregs (107). METTL14 deletion results in Sema4D accumulation, which represses PAK-STAT5 signaling, decreases FoxP3 expression, and ultimately leads to Treg dysfunction and accelerated allograft rejection (107).

Among various T helper (Th) cell subsets, METTL3-mediated m6A modification broadly influences CD4+ T cell fate and function (64). Follicular helper T (Tfh) cells, a specialized subset of CD4+ Th cells, have differentiation that is critically dependent on the METTL3-mediated m6A post-transcriptional regulatory pathway (159). Conditional knockout of METTL3 in CD4+ T cells significantly impairs Tfh cell proliferation and survival (159). Mechanistically, METTL3 catalyzes m6A modification on the 3’UTR of Tcf7 mRNA, stabilizing Tcf7 transcripts and maintaining TCF-1 protein expression, which drives Tfh cell differentiation (159). Evidence, both *in vitro* and *in vivo*, shows that T cell-intrinsic METTL3 deficiency markedly impairs Th17 cell differentiation, without noticeably affecting the differentiation of Th2 or Treg cells (64, 159).

#### Intrinsic regulation of T cell immune checkpoints

Programmed death-1 (PD-1) is a key inhibitory receptor that mediates T cell exhaustion. METTL14 directly catalyzes m6A modification on PDCD1 mRNA, which encodes PD-1 (110). The degradation of PDCD1 mRNA is mediated by YTHDF1/2/3, which sustains low PD-1 expression levels in T cells, thereby reducing T cell exhaustion and improving antitumor immunity (110). METTL14 deficiency results in elevated PD-1 levels, impairs CD8+ T cell activation, and accelerates tumor growth (110). This illustrates the bidirectional regulatory role of T cell-intrinsic METTL14: it maintains Treg suppressive function, indirectly exacerbating CD8+ T cell exhaustion, while also downregulating PD-1 expression in CD8+ T cells to protect their antitumor functions. The ultimate immune effect of METTL14 is contingent on the relative m6A modification activities across distinct T cell subsets within the TME.

### Macrophage-intrinsic METTL functions

Tumor-associated macrophages (TAMs) are the most abundant innate immune cells within the TME and exhibit substantial plasticity, enabling polarization into antitumor M1 or protumor M2 phenotypes. Macrophage-intrinsic METTLs modulate macrophage polarization, cellular metabolism, and immune function, thereby remodeling tumor immune responses.

METTL3 contributes to protumor M2 polarization in TAMs through its m6A methyltransferase activity (160). Increased macrophage METTL3 levels within the TME lead to hypermethylation and upregulation of the RBM14 protein via the m6A-dependent YTHDF1 axis, promoting macrophage M2 polarization and facilitating HCC progression (69). The m6A modifier ZC3H13 can regulate the nuclear transport of the METTL3/14 complex and stabilize CXCL8 mRNA, further supporting M2 polarization of TAMs (104). Conversely, lipoxin A4 (LXA4) secreted by prostate tumor cells downregulates METTL3 expression in TAMs, activating the STAT6 signaling cascade and inducing M2 polarization; restoration of METTL3 in macrophages can revert this protumor phenotype (154). Notably, METTL3 displays functional heterogeneity in TAMs. Research indicates that METTL3 can prevent TAMs from exhibiting a mixed M1/M2 polarization phenotype and promotes antitumor functions (161). Macrophage-specific deletion of METTL3 significantly diminishes the therapeutic efficacy of PD-1 checkpoint blockade, suggesting that METTL3 expression in macrophages directly influences the response to immunotherapy (161).

### NK cell-intrinsic METTL functions

NK cells are central innate antitumor effector cells that mediate tumoricidal activity through perforin (PRF1) and granzyme B (GZMB). NK cell-intrinsic METTLs post-transcriptionally modulate NK cell activation, proliferation, and cytotoxicity via m6A modification. As core components of the m6A methyltransferase complex, METTL3 and METTL14 demonstrate functional synergy and developmental stage specificity (161–164).

METTL3 plays a vital role in the homeostasis and antitumor capacity of mouse NK cells (162). Within the TME, TGF-β downregulates NK cell-intrinsic METTL3, resulting in a total decrease in m6A levels and suppression of NK cell effector functions. Specific knockout of METTL3 in NK cells leads to reduced NK cell populations, diminished tumor infiltration, impaired secretion of perforin, granzyme B, and IFN-γ, and exacerbated tumor metastasis. Mechanistically, m6A modification catalyzed by METTL3 contributes to the stabilization of SHP-2 protein expression and regulation of IL-15-downstream AKT/mTOR and MAPK/ERK signaling pathways. After METTL3 ablation, NK cells demonstrate attenuated IL-15 responses alongside defective proliferation, survival, and effector molecule production, although STAT5 phosphorylation remains unaffected, indicating that METTL3 predominantly regulates the AKT/MAPK signal cascade.

Humanized systems further confirm the positive regulatory role of METTL3 (163). In a human embryonic stem cell-derived NK cell model, METTL3 knockdown reduces the production of mature NK cells and downregulates T-BET and EOMES expression. This leads to decreased transcription and protein levels of PRF1, GZMB, and IFNG, resulting in weakened antitumor activity.

NK cell-intrinsic METTL14 exerts stage-dependent regulatory effects, primarily in early NK cell development (164). Conditional ablation of METTL14 in NK progenitor cells (NKPs) impedes their differentiation into immature NK cells and causes significant NK cell depletion. Interestingly, residual METTL14-deficient NK cells become hyperactivated and exhibit enhanced antitumor activity in melanoma models. Mechanistically, METTL14 stabilizes SOCS3 expression via m6A modification to suppress excessive activation of JAK-STAT5; downregulation of SOCS3 leads to hyperactivation of IL-15/JAK/STAT5 signaling and subsequent NK cell hyperactivation. In mature NK cells, METTL14 deletion does not disrupt NK cell homeostasis or effector capacity, highlighting its role as an early developmental checkpoint molecule.

Both NK cell-intrinsic METTL3 and METTL14 exhibit functional synergy (72). Deletion of either METTL3 or METTL14 alone minimally impacts NK cell homeostasis, while double knockout significantly disrupts NK cell maturation, homeostasis, and antitumor immunity. These protective functions of METTL3 and METTL14 are observed solely in normal NK cells. In contrast, METTL3 displays oncogenic effects in NKT lymphoma tumor cells (165, 166), reflecting abnormal expression following malignant transformation.

### Effects mediated indirectly through tumor metabolism

Tumor metabolic reprogramming serves as a core hub that remodels the TME and sustains tumor immune evasion. METTL-mediated m6A RNA modification alters tumor metabolic characteristics by controlling glucose and lipid metabolism, energy metabolism, and cell death pathways. Additionally, it mediates immune cell functions via metabolic intermediates, leading to overall immune remodeling of the TME and the establishment of a cascade regulatory network referred to as METTL-RNA modification-metabolic reprogramming-immune phenotype switch.

In this regulatory axis, tumor cell-intrinsic METTLs initiate metabolic reprogramming through m6A modification to reshape cellular metabolic patterns, subsequently altering the levels of metabolites such as lactate and cholesterol in the TME. These metabolites act as upstream signals that further regulate the activation, polarization, and functional states of infiltrating immune cells, ultimately contributing to the formation of an immunosuppressive microenvironment. Studies on non-alcoholic fatty liver disease-related HCC indicate that METTL3, through its m6A catalytic activity, enhances the translation efficiency of SCAP and other lipid metabolic genes, leading to increased tumor cholesterol secretion (61). Elevated cholesterol levels in the TME directly impair CD8+ T cell cytotoxicity (61).

Within the glycolysis pathway, tumor cell-intrinsic METTL3 and METTL14 promote augmented glycolysis, resulting in substantial lactate accumulation in the TME (62, 111). Lactate induces lactylation of METTL3 and activates the JAK1/STAT3 pathway, enhancing the immunosuppressive capacity of tumor-infiltrating myeloid cells and inducing CD8+ T cell exhaustion (62, 111). Additionally, lactate promotes M2d macrophage polarization characterized by high secretion of IL-10 and VEGF-A, further exacerbating immune evasion and vascular abnormalities (103). Concurrently, TME lactate upregulates macrophage METTL3 expression and enhances M2 polarization via OAS3 m6A modification (103). Polarized macrophages then release IL-6 to initiate the STAT3 cascade in tumor cells, leading to the upregulation of METTL3 and SLC16A1-AS1, thus establishing a METTL3-dependent metabolism-immune positive feedback loop (167).

The METTL family also remodels the TME by modulating ferroptosis metabolic pathways. In tumor cells, METTL5 and METTL16 stabilize antioxidant and ferroptosis-suppressive genes through m6A modification, resulting in reduced levels of tumor ferroptosis, resistance to CD8+ T cell cytotoxicity, and driving immunotherapy resistance (39, 82, 168). In certain pancreatic cancers, METTL3 activates RNA sensing pathways and promotes ferroptosis-related stress to reverse tumor immunosuppressive states (169).

In summary, METTL-mediated RNA modification regulates glucose metabolism, lipid metabolism, and ferroptosis metabolic networks, subsequently influencing immune cell polarization, exhaustion, and functional remodeling.

### Effects dependent on cytokines/hypoxia

Apart from metabolic reprogramming, hypoxia within the TME and cytokine signaling act as critical upstream switches regulating METTL-mediated m6A modification, thereby coordinating metabolic and immune pathways to construct the TME regulatory network. Microenvironmental signals, such as hypoxia, TGF-β, and IL-2/IL-15, bidirectionally influence tumor metabolism, immune cell homeostasis, and antitumor immunity by altering METTL expression and modification levels.

TGF-β serves as a core immunosuppressive factor in the TME, suppressing NK cell effector functions by downregulating METTL3 expression in NK cells (162). TGF-β facilitates Treg differentiation (170), while METTL3 and METTL14 are essential for maintaining Treg immunosuppressive phenotypes (171). IL-2, IL-12, and IL-15 are critical signals for activating NK cells and effector T cells. These cytokines activate the mTORC1/c-Myc/MAT2A axis, increasing intracellular SAM levels, facilitating METTL3/14-mediated m6A modification, and enhancing NK cell antitumor cytotoxicity (72).

TME hypoxia represents a canonical physicochemical characteristic of solid tumors, broadly regulating METTL expression in tumor and immune cells via HIF-1α-related pathways. In NK cells, hypoxia downregulates METTL3 and METTL14 expression, reduces m6A modification levels, and blocks the expression of effector molecules such as PRF1 and GZMB, thereby impairing NK cell antitumor functions (72). Furthermore, hypoxia can indirectly contribute to an immunosuppressive microenvironment by regulating the expression of chemokines and metabolic enzymes in tumor cells (172).

### Tumor type-specific effects

Tumors originating from distinct tissues exhibit diverse genomic backgrounds, TME compositions, and cellular metabolic characteristics, leading to significant tumor-type heterogeneity in the immunoregulatory effects of METTLs. The same METTL may exert opposing regulatory effects in different tumors, depending on tumor cell-intrinsic m6A readers and target RNAs.

The regulation of the PD-L1/PD-1 axis exemplifies this variability. In bladder, breast, and renal cell carcinomas, METTL3 enhances tumor immune evasion via its m6A methyltransferase activity, stabilizing PD-L1 mRNA through m6A modification and interaction with IGF2BPs, while also activating PI3K/AKT oncogenic pathways to upregulate PD-L1 transcription. These events suppress CD8+ T cell activation, promote T cell exhaustion, and cause immune evasion (40, 65–67). In glioblastoma, METTL14-mediated m6A modification on PD-L1 mRNA, recognized by IGF2BP2, enhances PD-L1 mRNA stability and expression, increasing proliferative, invasive, and metastatic capacities while promoting immune evasion (108). In hematological malignancies, METTL16 modifies the 3’UTR of PD-L1 mRNA via m6A methyltransferase activity, with subsequent recognition by IGF2BP2 leading to PD-L1 upregulation and resulting in tumor cell immune evasion and resistance to anti-PD-L1 immunotherapy (173). In pancreatic cancer, METTL3 contributes to immune evasion by stabilizing PD-L1 mRNA (66). However, recent evidence indicates broad downregulation of METTL3 in pancreatic ductal adenocarcinoma, with reduced METTL3 expression correlating positively with malignant clinical phenotypes (169). This seemingly contradictory finding suggests that METTL3 exhibits bidirectional immunoregulatory effects by regulating distinct downstream targets and activating multiple independent pathways, even within the same tumor type. In non-small cell lung cancer and gastric cancer, METTL3-dependent m6A modification of PD-L1 mRNA promotes YTHDF2 recruitment (60, 68), which accelerates PD-L1 mRNA degradation and downregulates protein levels, ultimately resulting in local tumor immunosuppression (60, 68).

### Context dependence and apparently opposing functions of METTLs

Accumulating evidence indicates that METTL family proteins exhibit context-dependent bidirectional regulatory characteristics within the TME, transcending simplistic classifications as merely oncogenic/tumor-suppressive or immune-enhancing/immune-suppressive. The seemingly contradictory findings in prior studies reflect not random literature discrepancies but rather a comprehensive manifestation of the intrinsic functional heterogeneity present within the METTL family. The ultimate biological effects arise from a multidimensional regulatory system that encompasses various determinants, including cell types, target RNA substrate signatures, intracellular m6A readers, downstream signaling cascades, tumor genetic profiles, tissue origins, physicochemical and inflammatory signals, experimental models, and treatment contexts.

Regarding cellular functions, METTL-mediated regulation exhibits strict subcellular compartmental specificity. A single METTL member can promote tumorigenesis and exert immunosuppressive effects in tumor cells, Treg cells, and macrophages, thereby facilitating tumor immune evasion. Conversely, in CD8+ T cells and NK cells, the same METTL may uphold immune cell homeostasis and enhance antitumor immune activity. This fundamental dichotomy between tumor cell-intrinsic and immune cell-intrinsic functions constitutes the core cellular basis for the bidirectional immune regulation exerted by the METTL family.

In terms of molecular mechanisms, METTL-mediated tumor immune regulation adheres to a unified hierarchical paradigm. Specific METTL proteins modify particular target transcripts and recruit distinct m6A readers to activate or silence downstream signaling pathways, ultimately shaping tumor type-specific immunophenotypes. Variations in molecular backgrounds and reader expression profiles across different tumors provide the fundamental molecular mechanisms through which the same METTL member can exert diametrically opposite regulatory effects.

Notably, the functional heterogeneity of the METTL family results in a significant clinical therapeutic paradox. While broad-spectrum systemic inhibition of METTL activity may suppress malignant progression in tumor cells, it simultaneously disrupts the physiological functions of CD8+ T cells and NK cells, diminishing host intrinsic antitumor immunity and potentially offsetting therapeutic benefits.

In summary, the tumor immune regulation mediated by METTL family proteins exhibits remarkable microenvironmental plasticity and spatiotemporal specificity. This characteristic highlights the limitations of traditional targeted inhibition strategies and necessitates that future translational research on METTL family proteins focuses on precise targeting based on cell types, tumor subtypes, and microenvironments. This approach provides new theoretical support for addressing tumor immune heterogeneity and optimizing personalized immunotherapy.

## METTL inhibitors

Numerous compounds targeting METTL family proteins have been reported to date; however, most exhibit low selectivity and significant toxicity risks, with only a few candidates advancing to clinical trials. This section systematically reviews the progress made in developing inhibitors that target METTLs (**Figure 4**; **Table 4**).

**Figure 4:**
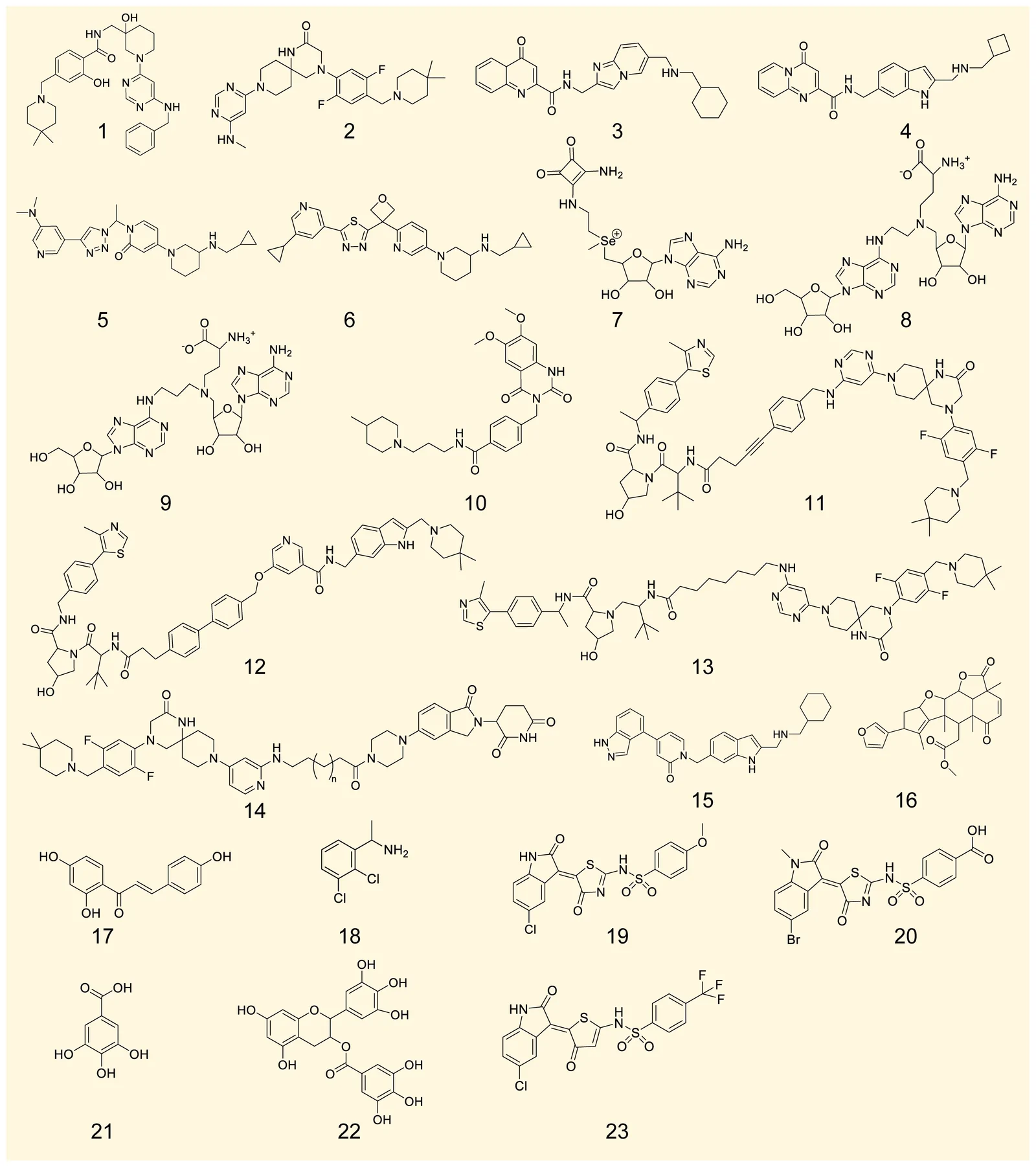
Representative inhibitors targeting METTL family members.

**Table 4:** Inhibitory activities of METTL inhibitors.

Compound number^a^	Compound name or chemical name	IC_50_/DC_50_/K_d_ value^b^	Reference
METTL3/14 complex inhibitor			
1	UZH1a	280 nM (IC_50_)	(174)
2	UZH2	5 nM (IC_50_)	(174)
3	STM2457	16.9 nM (IC_50_)	(175)
4	STC-15	6.1 nM (IC_50_)	(174)
5	EP652	2 nM (IC_50_)	(176)
6	EP102	2 nM (IC_50_)	(177)
7	Compound 11	9.75 nM (IC_50_)	(178)
8	Bisubstrate analogue 2	9 μM (IC_50_)	(179)
9	Bisubstrate analogue 4	18 μM (IC_50_)	(179)
10	C191-0266	7.64 μM (IC_50_); 7.28 μM (K_d_)	(180)
11	WD6305	0.14 µM (DC_50_)	(181)
12	AF151	0.43 µM (DC_50_)	(181)
13	ZW27941	0.13-0.7 µM (DC_50_)	(182)
14	ZW30441	0.44 µM and 0.13 µM (DC_50_)	(183)
	RSM3	3.10 µM (K_d_)	(184)
15	1-((2-((Cyclohexylmethyl)amino)methyl)-1H-indol-6-yl)methyl)-4-(1H-indazol-4-yl)pyridin-2(1H)-one	50 nM (IC_50_)	(185)
16	Nimbolide	0.10 μM (K_d_)	(186)
17	Isoliquiritigenin	*N.D.*	(187)
METTL7A inhibitor			
18	(±)-2,3-dichloro-α-methylbenzylamine	1.17 μM (IC_50_)	(129)
METTL16 inhibitor			
19	Aminothiazolone Hit compound 1	16.3 μM (IC_50_)	(188)
20	Aminothiazolone Hit compound 45	1.7 μM (IC_50_)	(188)
21	Gallic acid	37.2 μM (IC_50_)	(189)
22	Epigallocatechin-3-gallate	6 μM (IC_50_)	(189)
23	CHEMBL5597547	*N.D.*	(190)

^a^as shown in **Figure 4**; ^b^*N.D.* denotes not detected.

### METTL3/14 complex inhibitor

METTL3 and METTL14 typically form a stable heterodimeric complex that mediates mRNA m6A modification (23). METTL3 possesses primary methyltransferase activity, responsible for transferring a methyl group from SAM to the N6 atom of adenine (24). Although METTL14 lacks independent catalytic activity, it ensures precise m6A site distribution by recognizing RNA substrate sequences. Disruption of this complex abolishes m6A modification, presenting potential avenues for tumor intervention.

Early studies primarily centered on the development of SAM-competitive small-molecule inhibitors. Notable compounds, UZH1a and UZH2, exhibit *in vitro* IC_50_ values of 280 nM and 5 nM, respectively, for inhibiting METTL3 catalytic activity (174). UZH1a dose-dependently reduces m6A methylation levels in MOLM-13, U2OS, and HEK293T cells, demonstrating selectivity against several RNA methyltransferases (174). Although UZH2 effectively and selectively decreases the m6A/A ratio in PC-3 and MOLM-13 cells, its impact on MOLM-13 cell proliferation is minimal (191). These findings suggest that sole catalytic pocket inhibition may not yield optimal antitumor efficacy in certain tumor models, indicating that future development should also address non-catalytic functions or protein degradation strategies. In 2021, Yankova et al. reported STM2457, a selective candidate inhibitor of the METTL3/14 complex, with an IC_50_ of 16.9 nM (174, 192). STM2457 competitively binds to the SAM pocket with a Kd value of 1.4 nM (192) and exhibits significant *in vitro* antiproliferative activity against human adrenergic neuroblastoma cells (IC_50_ = 8.27-25.12 µM) and drug-resistant acute myeloid leukemia (AML) cells (IC_50_ = 3.37 μM) (175, 187, 193). Additionally, it synergizes with BET inhibitors to induce apoptosis in ovarian cancer cells (194) and inhibit anoikis (195). STC-15 (also known as STM3480) from STORM Therapeutics is the first pyrimidinone-scaffold METTL3/14 candidate inhibitor undergoing clinical studies (196). STC-15 inhibits the catalytic activity of the METTL3/14 complex with an IC_50_ of 6.1 nM (174). A Phase I/II clinical trial, initiated in December 2022, evaluates this compound in various advanced malignancies as monotherapy or in combination with toripalimab (**Table 5**). Preliminary results indicate the tolerability of oral administration, with adverse events such as thrombocytopenia and pruritus reported (NCT05584111) (197, 198). However, this trial primarily assesses safety, tolerability, and preliminary efficacy signals, and no confirmed antitumor clinical benefit has been documented. Dutheuil et al. developed EP652 using quantitative structure-activity relationship models to enhance oral bioavailability; it represents a new generation of selective candidate inhibitors for the METTL3/14 complex with an IC_50_ value of 2 nM. Low-dose EP652 (3 mg/kg) induced over 80% RNA m6A inhibition, with efficacy surpassing that of 30 mg/kg STC-15 (176). EP102, developed through further structural optimization (177), has demonstrated increased oral bioavailability and antitumor efficacy in animal models bearing AML and ovarian cancer, while maintaining enzyme inhibitory activity (177). EP102 is currently in the clinical research phase for advanced solid tumors (NCT07163325). Du et al. designed and synthesized a series of SAM analogs, with compound 11 showing potent inhibitory effects against the METTL3/14 complex (IC_50_ = 9.75 nM) (178). This compound exhibited no significant inhibition of METTL16 or DNA/tRNA methyltransferases, demonstrating promising *in vitro* selectivity (178). To address the limitations of traditional single-site SAM-competitive inhibitors, Corbeski et al. designed bisubstrate analogues (BAs) that covalently link SAM analogues with adenosine, allowing for simultaneous occupancy of both the SAM and adenosine pockets of METTL3 (179). Compounds BA2 and BA4 mimic the methyl-transfer transition state, enhancing inhibitory efficiency and affinity for the METTL3/14 complex, with IC_50_ values of 9 μM and 18 μM, respectively (179, 199). Although optimization of their catalytic activity is necessary, this strategy offers a new direction for developing METTL-targeted molecules.

**Table 5:** METTL-targeting agents under clinical investigation.

METTL inhibitors	Trial phase	Indication	Sponsor	Trial ID	Recruitment status	Verification date
EP102	Phase I	Advanced solid tumor	Epics Therapeutics	NCT07163325	Recruiting	2026-06
METTL3 peptide inhibitors	–	Urinary system tumors (bladder cancer, prostate cancer, kidney cancer)	Xijing Hospital	NCT06762925	Not yet recruiting	2024-12
STC-15	Phase I	Advanced solid tumor	STORM Therapeutics LTD	NCT05584111	Completed	2025-03
STC-15	Phase I/II	Solid tumor, sarcoma, leiomyosarcoma, dedifferentiated liposarcoma	STORM Therapeutics LTD	NCT06975293	Recruiting	2026-08

Early-phase clinical trials are designed to evaluate safety, tolerability, pharmacokinetics, and preliminary pharmacodynamic activity. Clinical benefit has not yet been established. Clinical-trial information is subject to real-time updates from ClinicalTrials.gov (https://clinicaltrials.gov/).

The quinazolinone-scaffold molecule C191–0266 was identified by Hu et al. through convolutional neural networks and deep learning virtual screening (180). *In vitro* biochemical experiments showed that C191–0266 can bind to the catalytic pocket located nearby the METTL3 SAM, with K_d_ = 7.28 μM and IC_50_ = 7.64 μM, alongside acceptable pharmacokinetic and safety characteristics (180). Cell experiments have confirmed that this compound can reverse cytarabine resistance in leukemia cells (180). This work demonstrates the drug development approach that transitions from computational screening to biochemical and cellular functional verification.

Although SAM-competitive inhibitors can rapidly suppress enzymatic catalytic activity, they possess inherent limitations. Blocking the catalytic function of METTL3 alone does not disrupt the protein-protein interactions between METTL3 and METTL14. The proteolysis-targeting chimera (PROTAC) strategy emerges as a next-generation therapeutic intervention capable of inducing complete degradation of target proteins via the ubiquitin-proteasome pathway. This approach can eliminate both catalytic and non-catalytic oncogenic functions of METTL3. Utilizing the UZH2 scaffold, Weldert et al. developed the METTL3-PROTAC WD6305 (181), followed by the synthesis of AF151 based on the STM2457 indole-nicotinamide scaffold (181). Both compounds efficiently degrade METTL3 protein, with half-degradation concentration (DC_50_) values of 0.43 µM and 0.14 µM, respectively (181). AF151 inhibits leukemia cell proliferation with antiproliferative potency 2–4 times greater than that of STM2457 (181). Additionally, AF151 significantly reduces cellular m6A levels and exhibits a synergistic antitumor effect with vincristine (181). ZW27941, a VHL-recruiting METTL3-PROTAC developed by Nar et al., also utilized the UZH2 scaffold (182). ZW27941 selectively degrades METTL3 with high potency (DC_50_ = 0.13-0.7 µM), reduces total cellular m6A levels in leukemia cells, and demonstrates *in vitro* anti-leukemic activity superior to UZH2. This PROTAC induces cell cycle arrest and apoptosis, generating synergistic antitumor effects with cytarabine (182). Smith et al. introduced the CRBN-recruiting METTL3/14-PROTAC ZW30441 (183), which simultaneously degrades METTL3 (DC_50_ = 0.44 µM) and METTL14 (DC_50_ = 0.13 µM), leading to decreased cellular m6A levels. Its *in vitro* anti-leukemic activity surpasses that of UZH2 and STM2457; however, ZW30441 suffers from inadequate metabolic stability, necessitating further optimization of the linker region and related structures (183).

In addition to catalytic inhibition and protein degradation, directly disrupting METTL3-METTL14 interactions has emerged as a promising strategy. Li et al. developed RSM3, the first stapled peptide inhibitor targeting the METTL3-METTL14 interaction (184). RSM3 exerts antitumor effects through two mechanisms: disrupting complex formation and inducing METTL3 degradation, thereby completely inhibiting m6A modification (184). This compound has a Kd value of 3.10 µM and reduces METTL3 enzymatic activity by 45.5% (184). Notably, RSM3 demonstrates superior antitumor effects in solid tumor models, such as prostate cancer, compared to STM2457 (184), suggesting that interference with protein interactions holds potential advantages for treating solid tumors. Peptide-targeting molecules are also advancing toward clinical exploration. A clinical trial for a METTL3 peptide inhibitor (NCT06762925) has been initiated by Xijing Hospital for urogenital tumors, though recruitment has yet to commence. The *in vivo* pharmacokinetic properties, selectivity, and human safety of such candidates require further evaluation.

Zhang et al. utilized scaffold hopping, concept restriction, and fragment growth strategies to design and synthesize a series of novel METTL3 inhibitors featuring a pyridin-2(1H)-one moiety (185). Among these, compound 15 demonstrated an IC_50_ value of 50 nM, inhibiting METTL3 catalytic activity three times more effectively than STM2457. It also exhibited favorable metabolic stability in human and mouse liver microsomes, with half-lives of 49.8 and 40.8 minutes, respectively, and showed superior antiproliferative activity in MOLM13 leukemia cells (185).

Natural small-molecule compounds are emerging as significant supplements to METTL3 inhibitors. Nimbolide has exhibited broad-spectrum antitumor activities both *in vivo* and *in vitro* (200–202). Cai et al. confirmed that nimbolide specifically inhibits METTL3 (186). Molecular docking and molecular dynamics simulations predicted that nimbolide can stably bind to the active pocket of METTL3, yielding a K_d_ value of 0.10 μM. Subsequent cellular experiments indicated that it has a preference for METTL3, with relatively minor effects on METTL14, FTO, and ALKBH5 (186). It is noteworthy that this binding affinity has not yet been directly measured by *in vitro* protein biochemical experiments, and the actual binding mode and selectivity of nimbolide still require further biochemical verification. Additionally, Jiao and colleagues identified isoliquiritigenin from a natural compound library, demonstrating high affinity for the catalytic domain of METTL3 (187). Its antiproliferative effects on MOLM13 and THP-1 leukemia cells (IC_50_ = 7.43 µM and 10 µM, respectively), coupled with superior pro-apoptotic activity compared to STM2457, suggest its potential as a METTL3 inhibitor for leukemia treatment (187). Natural small-molecule compounds often possess scaffolds distinct from fully synthetic small molecules, offering opportunities to address issues of inadequate selectivity and drug resistance associated with existing therapies.

### METTL7A inhibitor

METTL7A functions as either a small-molecule thiol methyltransferase or an RNA methyltransferase (35, 129). It accelerates tumor invasion and metastasis through m6A-dependent regulatory pathways (35, 129). Research on small-molecule inhibitors targeting METTL7A remains in its early stages. One of the few validated METTL7A-specific candidate inhibitors is (±)-2,3-dichloro-α-methylbenzylamine (DCMB). Russell et al. demonstrated that DCMB strongly binds to the catalytic domain of METTL7A and inhibits its methyltransferase activity, exhibiting an IC_50_ value of 1.17 μM and showing no significant inhibitory effects on METTL7B (129). Further studies indicated that DCMB effectively blocked METTL7A-mediated methylation modifications of substrates (129). However, despite the conserved catalytic domains and shared SAM methyl donor between METTL7A and METTL7B, direct experimental evidence confirming whether DCMB suppresses METTL7A-mediated RNA m6A modification remains lacking.

### METTL16 inhibitor

METTL16 drives tumorigenesis through both m6A-dependent and -independent mechanisms, establishing itself as an emerging target for tumor-specific therapy (29, 118, 203–205). Inhibitors targeting METTL16 can be categorized into those that target the SAM pocket and those that target the RNA-binding pocket. The former inhibits METTL16’s catalytic function, while the latter impairs m6A catalysis and disrupts the non-catalytic RNA-binding function of METTL16. Early METTL16 inhibitors focused primarily on the SAM-binding pocket, which posed challenges in achieving selective inhibition. However, since 2024, significant advancements in screening technologies and structural analysis of the target protein have facilitated the development of METTL16-targeted inhibitors.

To minimize off-target effects associated with SAM-competitive inhibitors, Liu et al. from the Max Planck Institute introduced a novel strategy focusing on the RNA-binding pocket of METTL16 (188). The sequence and structural features of this pocket differ significantly among various methyltransferases, rendering it an ideal target for optimizing selectivity. Through fluorescence polarization-based high-throughput screening of 25,000 small molecules, Liu et al. identified the first aminothiazolone-derived hit compound 1, which exhibited an IC_50_ of 16.3 μM for inhibiting METTL16 enzymatic activity. Following systematic structural optimization, the team synthesized 49 derivatives, ultimately yielding the highly active lead compound 45 (IC_50_ = 1.7 μM). This compound binds reversibly and non-covalently to the RNA-binding pocket of METTL16, directly occupying the RNA-binding site and disrupting METTL16 interactions with various RNA substrates, including MAT2A mRNA, U6 snRNA, and precursor miRNAs. Cellular experiments demonstrated that the inhibitor suppressed colony formation in TNBC cells and modulated overall intracellular m6A modification and MAT2A splicing. This study validates the therapeutic potential of targeting the RNA-binding pocket, laying a foundation for the development of m6A-related METTL16 inhibitors.

Progress has also been made with natural product-derived covalent METTL16 inhibitors. In 2026, Zhang et al. utilized reverse activity-based protein profiling to screen natural products and a library of approved probes (189). Based on the active protein probe experiment, it was found that gallic acid (GA) can covalently bind to K252 and C253 residues within the METTL16’s RNA-binding groove. *In vitro* enzymatic activity assays determined that GA suppresses the methyltransferase activity of METTL16 (IC_50_ = 37.2 μM) and represses METTL16-mediated pro-translational effects. Epigallocatechin-3-gallate (EGCG) with enhanced activity was identified following structure-activity relationship optimization. Biochemical assays indicated that the *in vitro* METTL16 enzymatic IC_50_ of EGCG is 6 μM, approximately six-fold higher than the inhibitory activity of GA. This molecule also targets K252/C253 residues and suppresses A549 cell proliferation and colony formation. Given that EGCG is a well-studied natural component with validated drug-like properties, this finding supports the potential for rapid clinical translation of METTL16 inhibitors.

Additionally, virtual screening has aided in the discovery of METTL16 candidate inhibitors. In 2026, Li et al. identified a series of selective METTL16 inhibitors from the ChEMBL database (190). Among these, CHEMBL5597547 demonstrated the strongest binding affinity for METTL16 (Vina score = -7.8) and has undergone preliminary drug-likeness evaluations, pending further functional validation.

Despite the progress made, current METTL16 candidate inhibitors exhibit multiple limitations. Some compounds demonstrate poor cellular activity, while covalent inhibitors require additional validation of tissue selectivity, all of which will necessitate refinement in future research.

## Discussion

METTLs play critical roles in post-transcriptional regulation, post-translational modification, chromatin remodeling, and the DNA damage response, and they are broadly implicated in tumor progression, metastasis, and drug resistance. Members of the METTL family can reprogram the TME and remodel immune cell functions by modulating m6A modification on immune-related molecules, ultimately influencing tumor immune surveillance and evasion. This review systematically outlines the structural characteristics and tumor biological functions of METTLs, with particular emphasis on their distinct regulatory mechanisms in immune cells such as CD8+ T cells, Tregs, TAMs, and NK cells. Additionally, this review summarizes recent advancements in inhibitors targeting METTLs and their translational prospects.

While existing studies establish METTLs as promising targets for regulating tumor immunity, several key scientific challenges remain unaddressed, and a considerable gap persists between current targeted interventions and mature clinical applications. First, current research predominantly focuses on METTL3, METTL14, and METTL16, whereas the immune functions and regulatory networks of less-studied members (i.e., METTL4, METTL5, METTL7, and METTL17) lack systematic analysis. Second, METTLs exhibit bidirectional and context-dependent regulatory effects across different tumors. The central mechanisms driving functional differences due to tumor subtypes, gene mutations, target RNA specificity, and TME heterogeneity require further elucidation. Third, existing METTL inhibitors generally exhibit significant deficiencies, such as inadequate selectivity, pronounced off-target effects, and low metabolic stability. The high homology of catalytic domains among METTL family members complicates the development of highly selective pharmacological agents.

Moreover, METTLs face multiple bottlenecks in the translational application of tumor immunotherapy. One critical issue is the unclear immunological safety associated with systemic METTL inhibition. Given that METTL3 and METTL14 play roles in maintaining normal immune cell physiological functions, systemic drug intervention might weaken the antitumor activity of natural T and NK cells, potentially leading to adverse therapeutic effects. Additionally, the therapeutic threshold for m6A modification has yet to be quantified, leaving the effective antitumor levels and safe ranges that help maintain immune homeostasis undetermined. Furthermore, the limited selectivity and significant off-target effects observed in current inhibitors, coupled with unassessed hematopoietic toxicity, could pose risks to hematopoietic homeostasis. Cell-type targeting differences also remain poorly understood. METTLs may function oppositely in tumor and immune cells, and the optimal cellular targets and intervention strategies for effective tumor immunotherapy remain to be clarified. Additionally, simple catalytic inhibition is often inadequate. Given that METTLs possess both catalytic and non-catalytic functions, solely suppressing their catalytic activity fails to comprehensively block their oncogenic and immunoregulatory effects, partially explaining the limited therapeutic efficacy of various inhibitors. Lastly, the mechanisms underlying targeted drug resistance and compensatory pathways are still underexplored. It has yet to be established whether inhibition of m6A modification induces compensatory activation of other RNA modification pathways, which may confer subsequent therapeutic resistance.

Moreover, METTL-targeted candidate agents in clinical research continue to face numerous translational challenges. First, the high conservation of catalytic domains across the METTL family makes traditional small-molecule inhibitors susceptible to off-target effects. Second, METTLs are essential for the functioning of normal hematopoietic and immune cells; thus, pharmacological interventions may induce hematopoietic toxicity and disrupt normal immune responses, consequently narrowing the therapeutic window. Third, many current candidate molecules rely on systemic administration, which does not differentiate between tumor cells and immune cells *in vivo*, complicating efforts to avoid toxicities to normal tissues. Fourth, the absence of biomarkers for dynamically assessing METTL protein inhibition and m6A modification levels in patients limits the ability to screen potential beneficiaries and monitor clinical efficacy effectively. Fifth, therapeutic resistance can arise through various mechanisms, including compensatory activation of RNA modification pathways, target mutations, increased target protein expression, and the activation of bypass signaling cascades. Lastly, prolonged interference with physiological m6A and other RNA methylation modifications due to METTL inhibitors may disrupt normal stem cell homeostasis and lead to potential delayed toxicity.

The current research landscape also shows a limited understanding of spatial heterogeneity within the TME. Existing studies frequently overlook differences in spatial distribution within tumor tissues, making it challenging to accurately characterize the *in situ* expression profiles of METTLs across distinct locations and cell subpopulations, as well as the associated m6A regulatory patterns. Immune and tumor cells situated in the tumor parenchyma, invasive margins, stromal regions, and hypoxic necrotic areas exhibit notable spatial distribution heterogeneity, leading to region-specific variations in METTL-mediated m6A modification, metabolic reprogramming, and immunomodulation. Advanced technologies such as single-cell transcriptomics and spatial transcriptomics provide robust tools to address these challenges. Single-cell transcriptomics allows for precise analysis of METTL-specific expression profiles in distinct cell subtypes, avoiding the averaging effect common in pooled cell populations. Spatial transcriptomics, on the other hand, enables the localization of METTLs and m6A-related regulatory molecules, contributing to the construction of a METTL-m6A-metabolism-immune regulatory network. Employing these cutting-edge techniques, future studies can delineate cell-specific METTL functions in various spatial microenvironments and elucidate how spatial compartmentalization within the TME influences the bidirectional immune regulatory roles of METTLs.

To address the above challenges, future research should consider the following avenues for breakthroughs: (1) Utilize m6A-seq, transcriptomics, and CRISPR-Cas9 screening to systematically analyze the expression characteristics and immune regulatory mechanisms of METTLs across different tumors, filling the knowledge gaps regarding non-canonical METTL functions; (2) Investigate how tumor subtypes, microenvironmental metabolism, and signaling pathways affect the bidirectional functions of METTLs and establish a molecular typing framework to predict their immune modulatory effects; (3) Integrate structural biology and high-throughput screening to develop selective small-molecule inhibitors and PROTACs that target both catalytic and non-catalytic functions, thereby minimizing off-target effects; (4) Explore combinatorial treatment strategies that incorporate METTL inhibitors alongside immune checkpoint inhibitors, chemotherapy, or targeted therapies to mitigate immunosuppression and treatment resistance; (5) Develop prognostic and efficacy prediction models based on METTL expression profiles and m6A modification features to select appropriate patients and implement individualized, precision therapeutics.

Notably, the non-catalytic functions of METTLs are key factors influencing therapeutic strategies. For instance, METTL3’s oncogenic role relies on both its m6A catalytic activity and independent protein interactions. Inhibitors that target solely the catalytic pocket may demonstrate limited efficacy, while PROTAC degraders can comprehensively deplete the protein and disrupt both its catalytic and non-catalytic functions, offering enhanced therapeutic effects. Therefore, future translational research should accurately differentiate the functional dependencies of distinct METTLs and judiciously select between catalytic inhibition and protein degradation approaches to provide theoretical support for combination strategies in tumor immunotherapy.
